# Measuring the Closeness of Relationships: A Comprehensive Evaluation of the 'Inclusion of the Other in the Self' Scale

**DOI:** 10.1371/journal.pone.0129478

**Published:** 2015-06-12

**Authors:** Simon Gächter, Chris Starmer, Fabio Tufano

**Affiliations:** 1 Centre for Decision Research and Experimental Economics, School of Economics, University of Nottingham, University Park, Nottingham, United Kingdom; 2 CESifo Network, Munich, Germany; 3 IZA Network, Bonn, Germany; University of the Basque Country, SPAIN

## Abstract

Understanding the nature and influence of social relationships is of increasing interest to behavioral economists, and behavioral scientists more generally. In turn, this creates a need for tractable, and reliable, tools for measuring fundamental aspects of social relationships. We provide a comprehensive evaluation of the 'Inclusion of the Other in the Self' (IOS) Scale, a handy pictorial tool for measuring the subjectively perceived closeness of a relationship. The tool is highly portable, very easy for subjects to understand and takes less than 1 minute to administer. Across our three online studies with a diverse adult population (n = 772) we show that six different scales designed to measure relationship closeness are all highly significantly positively correlated with the IOS Scale. We then conduct a Principal Component Analysis to construct an Index of Relationship Closeness and find that it correlates very strongly (ρ = 85) with the IOS Scale. We conclude that the IOS Scale is a psychologically meaningful and highly reliable measure of the subjective closeness of relationships.

## Introduction

An important fact of social life is that people have social relationships that vary in closeness. Most people have some close relationship to a romantic partner, a few friends and family, somewhat looser relationships with other friends and even looser ones with numerous acquaintances. If such relationships are a fact of social life, how then do relationships matter for social behavior and social preferences? We suggest these important issues provoke a prior question of how the perceived closeness of a relationship, which is a subjective judgment, can be measured. In this paper, we hope to make some progress in answering this prior question by evaluating in three studies a simple pictorial measurement instrument, called the 'Inclusion of the Other in the Self' Scale, by Aron et al.[1]. The question, of how social relationships matter for social behavior, is the ultimate motivation underlying our interest, as behavioral scientists, in the measurement of relationships. Here is why.

For a long time many economists and other social scientists used the *homo economicus* assumption to explain social behavior. *Homo economicus* conceives of social relations purely from the point of view of an individual’s self interest, and from this perspective relationships only matter indirectly, to advance own well-being. Behavioral research in the last three decades has changed one aspect of this picture profoundly by demonstrating that many people have strong other-regarding preferences [2–5]. However, much of this research abstracts from the social relationships people actually have and measures social preferences towards *unidentified*, *anonymous* other people. Thus, relationship closeness is disregarded even if other-regarding motives matter strongly. Similarly, behavioral investigations of strategic thinking largely disregard the psychological nature of the relationships among individuals and, in experiments, usually only study anonymous agents and their interactions.

Thus, in our view, a significant open research question is how social preferences or strategic thinking change with the nature of real, non-anonymous relationships. Some evidence suggests that the degree to which individuals can identify each other matters for social preferences (e.g., [6], [7]), but this research does not directly investigate the role of relationships. Some contemporary research measures a concept akin to relationships, namely the network structures in which people are embedded (people are asked to list the names of their friends or people with whom they interact) and then this network structure is related to a variable of interest, e.g., altruistic sharing [8, 9] or diffusion of information [10]. These are excellent tools to advance our understanding of the importance of social relationships. However, network structures highlight who is linked to whom and who is "central" and do not consider the psychology of relationships, that is, how "close" people feel to be to a specific other person.

**Fig 1:**
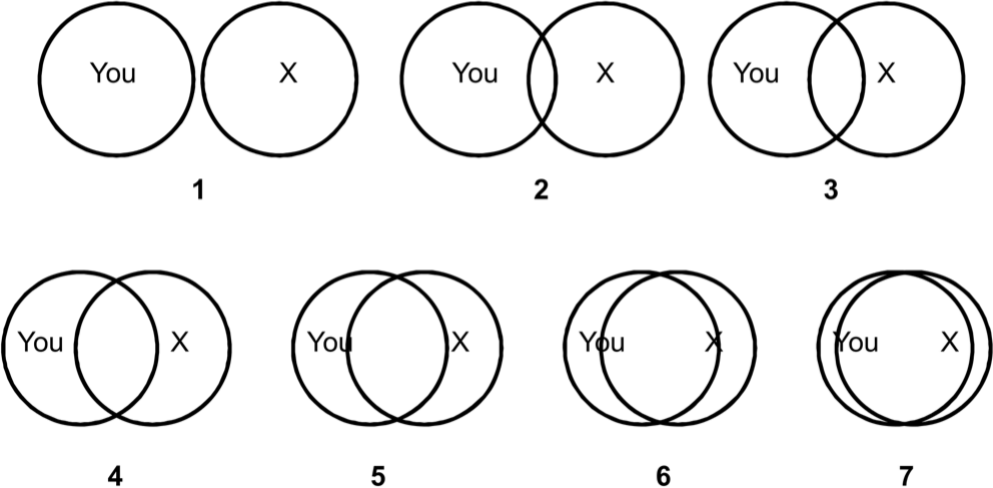
The 'Inclusion of the Other in the Self' (IOS) task. Adapted and changed for our online implementation from AAS ([1], Fig 1, p. 597). Respondents are asked to select the pair of circles that best describes their relationship with X. AAS speak of ‘Self’ and ‘Other’, whereas we use the terminology ‘You’ and ‘X’.

In this paper we are motivated by a complementary strategy for advancing our understanding of relationships, which involves borrowing tools from social psychology to *measure* the closeness of bi-lateral personal and social relationships between individuals. The tool is the "Inclusion of the Other in the Self" (IOS) Scale, developed by Aron, Aron and Smollan in a highly cited 1992 paper in the *Journal of Personality and Social Psychology* ([1], henceforth AAS). It is a simple pictorial tool, which is very easy to implement making it a potentially highly useful instrument. We illustrate our version of the IOS task in Fig 1 (exactly as it was seen by the participants of our three studies); further details are given in the next section. The IOS task asks respondents (“You” in our version) to assess their relationship with a specific individual (referred to as "X" in our figure) by selecting one out of seven pairs of increasingly overlapping circles. In each pair of circles, one circle refers to the respondent and the other circle to X. Respondents are asked to select the pair of circles that best describes their relationship with X. For example, if a respondent feels unrelated to X, it would be natural to select the first pair of still disjoint circles; if a respondent feels very close to X, he or she may choose the almost completely overlapping set of circles.

The IOS Scale is extremely simple to use and, we believe, highly intuitive. It takes less than a minute to administer. It is also neutral about the reason why people feel close or distant; it just measures an individual’s own subjective perception of their degree of closeness to another person. AAS ([1], p. 598) argue that the "IOS Scale is hypothesized to tap people's *sense* of being interconnected with another. That sense may arise from all sorts of processes, conscious or unconscious […] The IOS Scale is intended to capture something in the respondent's perception of a relationship that is consistent with many theoretical orientations." Our evaluation of the IOS Scale is inspired by Aron et al.'s intention that the IOS Scale is "consistent with many theoretical orientations" and therefore involves linking several conceptually different scales that all intend to measure relationship closeness.

Our evaluation of the IOS Scale is motivated by two developments since AAS. First, AAS, in their original evaluation of the IOS Scale, were mostly interested in measuring close relationships but for behavioral scientists non-close relationships such as friends and acquaintances are often also of substantial interest. In response to such demand, Starzyk, Holden, Fabrigar and MacDonald ([11], henceforth, SHFM) developed a new questionnaire, the 'Personal Acquaintance Measure' (PAM) that allows measuring all types of relationships, close and non-close. Hence, we will also evaluate how the IOS Scale is related to the PAM Scale and compare our results to those of SHFM. Second, AAS as well as SHFM relied on student subjects and paper-based questionnaires administered in the classroom. Many potential applications of this tool, however, will nowadays use non-student subject pools and/or internet-based data collection methods and these facts provide a central motivation for the new evaluation of the IOS Scale we report here: Do the psychometric properties of the IOS Scale extend to non-students and the use of an internet-based survey tool?

We report three studies; all conducted with the help of Amazon.com's labor market Mechanical Turk (MTurk). Participants on MTurk are typically older and come from much more varied backgrounds than undergraduates. Study 1 replicates AAS and the psychometric evaluation reported in that paper. One important claim from AAS is that the IOS Scale captures the essence of a much more complex and comprehensive scale, the influential Relationship Closeness Inventory (RCI) developed by Berscheid, Snyder and Omoto ([12], henceforth BSO). As part of the evaluation of the IOS Scale, Study 1 will therefore also implement the RCI. Our results confirm all psychometric properties of RCI found in BSO and AAS for the new and more diverse population from which we sample. Most importantly, we confirm AAS's conclusions about the validity of the IOS Scale.

The RCI is predominantly about "close" relationships, such as close friendships and in particular romantic relationships. However, many relationships that are of special interest to behavioral social scientists are not romantic and not particularly close, but still quite important; think of workplace relationships as an example. Studies 2 and 3 will, therefore, also explore the IOS Scale for non-close relationships. In particular, in addition to close relationships, Study 2 will elicit measurements on the IOS scale for two more categories of relationships: *friends* who are more than an acquaintance, but not a person's most intimate relationship; and *acquaintances* who are closer than strangers, but less close than friends. We find that the IOS measurements vary strongly and in the expected directions across the three classes of relationship.

Study 3 elicits the IOS measurements for close relationships, friends and acquaintances and compare them to results based on a set of conceptually related questionnaires, such as, again, the RCI, the Liking and Loving Scale by Rubin [13], and the PAM Scale. Details of the conceptual background and implementation of the RCI and PAM will be given in the introduction and methods sections of the respective studies. Our findings confirm the results from Studies 1 and 2 as well as the most important findings of SHFM with regard to the PAM Scale.

One central observation of Study 3 is that the IOS Scale is highly significantly and strongly correlated with several scales that all measure dimensions of relationship closeness: among them, the RCI, the Loving and Liking Scales, and the PAM Scale. In our final section we use the fact that several different measures of relationship closeness correlate with IOS to perform a Principal Component Analysis to derive an "Index of Relationship Closeness" (IRC). We show that IRC correlates very highly (ρ = .85) with IOS. We conclude, therefore, that the IOS Scale is measuring important aspects of relationship closeness in a compact and highly reliable way, which coupled with the simplicity and portability of the tool, renders it a very useful instrument for any researcher interested in measuring the closeness of relationships.

## Materials and Methods

The Research Ethics Committee of the School of Economics of the University of Nottingham approved this research. The research was conducted using Amazon Mechanical Turk (http://www.mturk.com). Mturk is a crowd sourcing internet-based market place that facilitates hiring of "workers" to do short online jobs (called a HIT—"Human Intelligence Tasks") [14–16]. MTurk is popular for conducting research because it has a vast subject pool, which is more varied than the typical undergraduate subject pool. Although it is not fully representative of the (American) population at large, it is more representative than purely undergraduate subject pools and provides a very cost effective route to large data sets [17–20]. And, most importantly, it provides quality data, according to several studies that compared MTurk samples to more traditional lab samples where experimental control is typically stronger than on MTurk [16, 19–21].

Informed consent was obtained online from all participants (before they finally decided to take part participants were told the following: "By accepting this HIT you give us informed consent that we can use your answers in anonymized form for research purposes only.").

After they had accepted our HIT, participants read the following introductory text: "In this HIT we will ask you to respond to a questionnaire on the nature of interpersonal relationships. Our interest is entirely scientific. All answers will be treated confidentially and will only be reported in aggregated statistical form. There are no right or wrong answers in this survey; we are only interested in your honest assessment. If you feel uncomfortable answering some questions you will have opportunities to select 'prefer not to answer' as an answer."

A total of 772 people, all recruited on MTurk and residents of the United States, participated in our three studies. The studies were conducted between February and July 2014. The average age of our participants was 34 years (s.d. 11) and about 46% were female. In contrast, the participants in our reference studies were undergraduates with an average age of about 19 years. Across the three studies, the percentage of females varied between 52% and 78%. After deciding to participate, subjects were directed to the survey questions on an external webpage. The survey was coded using the survey software Qualtrics (http://www.qualtrics.com/). The survey questions are available as part of the Supporting Information to this paper (S1 Text). Participants received a flat payment for participation (details are given in the description of studies).

Participants were first introduced to the topic of the research and told that it is about understanding the nature of relationships (see above). They were then asked to focus on one specific individual (the exact rules varied across the studies we report here). Participants were asked to identify this individual by the initial of their first name. Participants were then told the following:

"In the following figure we ask you to consider which of these pairs of circles best describes your relationship with [This Individual] in all questions that follow. In the figure "X" serves as a placeholder for [This Individual], that is, you should think of "X" being [This Individual]. By selecting the appropriate number please indicate to what extent you and [This Individual] are connected."

On the screen the IOS task was presented exactly as in Fig 1. Participants had to select the answer with a simple click but they could also indicate that they preferred not to answer.

We also elicited two more measures proposed in the literature to measure relationship closeness. First, following Cialdini et al. [22], right after eliciting the IOS Scale rating we added a "We Scale". The exact wording was as follows: "Please, select the appropriate number below to indicate to what extent you would use the term “WE” to characterize you and [This Individual]. Answers were on a 7-point scale (1 = "not at all"; 7 = "very much so "). The average between the IOS Scale and the "We- Scale" forms a measure that Cialdini et al. [22] call "oneness".

Second, following BSO, we also measure a "Subjective Closeness Index" (SCI), which is based on the answers to two questions:

1. "Relative to all your other relationships (both same and opposite sex) how would you characterize your relationship with X?"
2. "Relative to what you know about other people's close relationships, how would you characterize your relationship with X?"

Answers were on a 7-point scale, where 1 = "not close at all", and 7 = "very close". The SCI is simply the sum of scores.

The IOS Scale, the We Scale, and the SCI Scale are used in all three studies. Study 1 benchmarks them against the RCI; Study 2 looks for differences in levels of relationship closeness; and Study 3 investigates further benchmarks, in particular the PAM Scale. The raw data are available as Supporting Information file S1 Dataset (in Excel format).

We close our comprehensive evaluation of the IOS Scale with a novel Principal Component Analysis we use to derive an "Index of Relationship Closeness". This index will be our comprehensive benchmark for the IOS Scale.

## Study 1

The main purpose of Study 1 was to replicate the RCI and in addition to see whether the IOS Scale is correlated with RCI, the We Scale, and the SCI Scale and how the correlations and the scores of the scales compare between our study and previous studies [1],[12]. The two most important instruments of Study 1 are the RCI and the IOS Scale. The RCI provides the conceptual background to the IOS Scale and therefore we describe it in more detail now.

The conceptual foundations for RCI are due to Kelley et al. who argue that a close relationship is characterized by high “interdependence” [23], which manifests itself in interconnected activities, where people have frequent impact on each other; the degree of impact is strong; and impact is based on diverse activities people undertake together. Based on this conceptual framework, the RCI is a 69-item self-report to measure the frequency with which partners see each other; how many diverse activities partners undertake together, and the strength of influence a partner has on the respondent. Answers are then aggregated into three subscales (RCI Frequency, RCI Diversity, and RCI Strength) and a total RCI Scale. We describe the questionnaires used to measure the RCI and its subscales in the methods section.

The RCI takes about 10–15 minutes to complete and is often too detailed for many research purposes. Therefore, AAS developed the IOS Scale (as per Fig 1), to have a handy and compact instrument to measure relationship closeness. AAS argue that the IOS is not linked only to the RCI but is consistent with several approaches to relationship closeness in social psychology (a claim we will evaluate in Study 3). Based on previous work [24], AAS ([1], p. 598) argue that "in a close relationship the individual acts as if some or all aspects of the partner are partially the individual's own" and that "in close relationships the individual may perceive the self as including resources, perspectives, and characteristics of the other".

### Methods

We recruited 200 volunteers on MTurk who received a flat payment of $3 for a survey that took most participants about 30 minutes. Our sample size is in the same ball park as our comparison studies: AAS, [1] had 208 participants (Primary Study), and BSO [12] had 241 participants (in their main RCI study).

The survey consisted of two parts. Part I included, in this order, the IOS and two related questions, and the RCI. The order of questionnaires was the same as in Part I of AAS. Part II consisted of a set of hypothetical decision problems, which we do not report here. When answering Part I questions, participants were not informed about the nature of questions in Part II; hence, Part II cannot influence the responses to IOS and RCI. Answering the questions of Part I took about 15 minutes.

We used the exact same texts and questions as BSO and only changed any wording related to the fact that ours was an online implementation of BSO's questionnaire. (Their exact wording can be found in BSO [12], Appendix A, pp. 806–807). All participants read the following introductory text (p. 806, and S1 Text):

"We are currently investigating the nature of interpersonal relationships. As part of this study, we would like you to answer the following questions about your relationship with another person. Specifically, we would like you to choose the *one* person with whom you have the *closest*, *deepest*, *most involved*, *and most intimate relationship*, and answer the following questions with regard to this particular person. For some of you, this person may be a dating partner or someone with whom you have a romantic relationship. For others of you, this person may be a close, personal friend, family member, or companion. It makes no difference exactly who this person is as long as she or he is the one person with whom you have the closest, deepest, most involved, and most intimate relationship. Please select this person carefully since this decision will affect the rest of this study. With this person in mind, please respond to the following questions."

Participants were then asked to provide the initial of the first name of the person they had in mind (here, we will refer to this person as "X"). The initial they provided was automatically inserted into all questions that referred to this specific individual. Participants were then presented with the IOS task, in exactly the same way as illustrated in Fig 1, followed by the "We Scale". Participants were also asked about the gender and age of X, as well as their own age and gender, how long they had known X, and whether X is a family member, a friend, someone from work, or a romantic partner. After these introductory questions, participants proceeded to the core questions that constitute the RCI.

The first block of questions concern the *frequency* of interactions in the past week, where participants are asked to assess how many hours they had spent with X alone in the morning, the afternoon, and in the evening. The stated times are translated into minutes and then given a score between 1 and 10, where the score increases in the frequency of interactions. This score constitutes the *RCI Frequency* score.

The second block of questions measures the *diversity* of activities that the participant and X undertook together in the past week. Participants were presented with a list of 38 activities and asked to check all that apply. The number of joint activities is translated into a score between 1 and 10, where the score increases in the diversity of activities. This score is the *RCI Diversity* score.

The third block of 34 questions measures the *strength* of influence that X has on the participants on a range of attitudes, time spent with friends or relatives, financial expenditures, leisure activities etc. A set of questions in this block also asks to what extent X influences the participant's future plans in a variety of domains from vacation plans to marriage plans. Participants had to answer on a 7-point scale (1 = strongly disagree; 7 = strongly agree). The sum of scores (which theoretically can be between 34*1 = 34 and 34*7 = 238) was then given a *RCI Strength* score, which ranges between 1 and 10 (higher score means higher strength).

The *Total RCI* score is then simply the sum of the RCI Frequency, RCI Diversity and RCI Strength scores. By construction, the Total RCI score takes values between 3 and 30. All specific details of the scoring rules can be found in BSO ([12], Appendix B, p. 807).

### Results

Our main purpose in Study 1 is to compare the psychometric properties of the IOS Scale and the RCI Scale as reported by AAS [1] and BSO [12]), respectively, with our data. The nature of the relationship with X differs predictably between our sample and the much younger student samples of BSO and AAS, respectively. In our sample, 68% report their closest relationship to be a romantic one, whereas in BSO and AAS this was the case for only 47% and 44%, respectively. In our sample, family and friends constitute 10.5% and 16.5%, respectively. For many student participants in BSO and AAS, their closest relationship was a friend (36% and 37%, resp.) or a family member (14% and 16%, resp.). The longevity of close relations was also substantially bigger among our participants (around 9 years for non-family members; 32 years for family members; compared to about 4.5 years for non-family and 19 years for family among students), which is unsurprising given the age difference between our sample and the students of previous samples.

Next we compare the internal consistency of the scales [25]. We have two comparison sets here, BSO and AAS, because in their validation of the IOS Scale, AAS also replicated BSO. Internal consistency (as measured by Cronbach's α) is remarkably similar across the three studies and all scales. For example, for RCI Total, our α = .65; for BSO it was α = .62; and for AAS it was α = .66. See S1 Table for further details.

Given our particular interest in the IOS Scale the most important comparison for our purposes is to look at the correlations between the IOS Scale, the RCI Scale, and the SCI Scale. The comparison data are from AAS ([1], Table 1, p. 600). The top panel of Table 1 reports the results for our sample and the bottom panel documents the respective results of AAS. Before we look at correlations we notice that the mean of the IOS Scale is 5.30 (sd 1.41) in our data and 4.74 (sd 1.48) in the AAS data. Thus, our participants report somewhat closer relationships as measured by the IOS Scale than the student participants of AAS. The mean scores of all scales are a bit higher in our sample compared to AAS.

**Table 1:** Correlations among IOS Scale, RCI Scales, and SCI Scale.

Scale	1	2	3	4	5	6
**A: Our Study 1**						
**1. IOS scale**	-	.41**	.31**	.26**	.36**	.54**
**2. RCI Total**		-	.83**	.74**	.72**	.36**
**3. RCI Frequency**			-	.51**	.36**	.16**
**4. RCI Diversity**				-	.30**	.34**
**5. RCI Strength**					-	.33**
**6. SCI**						-
**Mean**	5.30	16.80	5.72	5.07	5.94	12.66
**SD**	1.41	4.26	2.16	1.59	1.85	1.83
**B: AAS**						
**1. IOS scale**	-	.22**	.09	.16*	.36**	.34**
**2. RCI Total**		-	.90**	.88**	.50**	.07
**3. RCI Frequency**			-	.71**	.18**	-.01
**4. RCI Diversity**				-	.27**	.08
**5. RCI Strength**					-	.26**
**6. SCI**						-
**Mean**	4.74	14.06	4.85	4.49	4.68	12.03
**SD**	1.48	5.52	3.12	2.17	1.58	1.68

The data from AAS are taken from their Table 1 ([1], p. 600).

** p < .01

* p < .05

More importantly, the IOS Scale is highly significantly positively correlated with each of the RCI Scales and the correlation is at least as strong as in AAS. The IOS Scale is also highly significantly positively correlated with the SCI Scale and more strongly so than in AAS (0.54 compared to 0.34). The correlations of the RCI Scales with each other are mostly of a similar magnitude comparing our results to those of AAS.

Our data also allow for a comparison with the correlations of RCI subscales found by BSO. The strengths of correlations of the RCI subscales in our sample are surprisingly similar in most comparisons both by relationship type and by gender of the respondent (S2 Table).

A further result concerns the correlation of the "We Scale" with the IOS Scale and the RCI scales. The correlation with the IOS Scale is 0.64; the correlations with RCI Total, Frequency, Diversity, and Strength, are 0.55, 0.40, 0.39, and 0.48, respectively. The correlation with the SCI Scale is 0.52. All correlations are significant at p<0.0001.

Our final correlational analysis looks at "oneness", a measure proposed by [22]. Oneness is the average of the IOS Scale and the We Scale. The correlations with RCI Total, Frequency, Diversity, and Strength are 0.54, 0.40, 0.36 and 0.46, respectively. The correlation with the SCI Scale is 0.58. All correlations are significant at p<0.0001. These correlations are somewhat stronger than with the IOS Scale alone (cf. Table 1).

We conclude this results section with a comparison of the levels of scores across the RCI subscales and by relationship type. One observation is that almost all scores are higher in our data than in BSO. This is not surprising given that our participants are older and therefore probably enjoy more mature relationships than the students of BSO. However, one qualitative feature is the same between our data and BSO: if the closest relation is romantic, scores are higher than if the closest relation is a friend or family member. The difference is highly significant for all RCI subscales and also for RCI Total, whereas the differences between friends and family are insignificant. Similar conclusions hold for the We Scale and the SCI Scale (S3 Table).

We do not find gender differences in RCI Total, RCI Frequency, RCI Strength, the IOS Scale, and the We Scale. Females have somewhat higher RCI Diversity (5.4 vs. 4.9) and SCI scores (13 vs. 12.4) and these differences are significant at p = 0.029 and p = 0.0250, respectively, two-sided t-tests. These gender differences are in contrast to BSO who find no differences in the RCI scores.

## Discussion

Study 1 was designed to re-run BSO and AAS, with the main purpose of probing the validity of the IOS Scale for a non-student population and an online measurement. The results are very encouraging and vindicate BSO and AAS whose findings are almost perfectly replicated. With the exception of level differences in scores, which are explicable due to the age differences in populations between our study and BSO and AAS, all other measures and psychometric properties are both qualitatively and quantitatively very similar to BSO and AAS.

Study 1 was about close relations, such as relations with romantic partners, or close friends or family. However, many interesting relations are outside romantic partnerships and close friendships, which raises the question how the IOS Scale fares with non-close relationships. In addition to close relationships, Studies 2 and 3 also look at non-close relationships, such as friends and acquaintances. If the IOS Scale would not vary appropriately across these three generic levels of relationships, its usefulness for assessing other than very close relationships would be compromised.

## Study 2

The purpose of Study 2 was to test the IOS Scale for three levels of relationship closeness: IOS of a very close person, IOS of a friend, and IOS of an acquaintance. As further checks we also include the We Scale and the SCI Scale. We considered this study to be an intermediate step before deciding whether a fully-fledged investigation of the variation of the IOS Scale is warranted, across different levels of relationships, with a whole battery of further checks.

### Methods

We recruited 120 participants on MTurk for this study. They received a flat fee of $0.20 upon completion of the survey, which took about 2 minutes.

We had three treatment conditions in this study, which varied only one crucial sentence and the necessary, but minor, subsequent adaptations to the text. The three variations attempted to manipulate the type of relationship people should think about: a very close person, a friend, or an acquaintance. For this purpose we amended the introductory text as documented for Study 1 slightly (all emphases as in the texts presented to participants):

1. *Close relationships*: This text was exactly the same as in Study 1.
2. *Friends*: "[…], we would like you to choose a person with whom you have *a good friendship*, *who is more than an acquaintance*, *but not your closest*, *or most intimate relationship*, and answer the following questions with regard to this particular person. For some of you, this person may be a personal friend. For others of you, this person may be a family member, or companion. It makes no difference exactly who this person is as long as she or he is a good friend, who is more than an acquaintance, but not your closest, or most intimate relationship."
3. *Acquaintances*: "[…], we would like you to choose a person whom you consider an *acquaintance*, *but no more than an acquaintance*, and answer the following questions with regard to this particular person. For some of you, this person may be a colleague at work. For others of you, this person may be a neighbor, or member of your wider social network. It makes no difference exactly who this person is as long as she or he is a person who you consider an acquaintance, but no more than an acquaintance."

Participants were then prompted to think about a specific person and to provide the initial of that person's first name. Participants did the IOS task and also answered the "We question", and the SCI Scale, in that order. The allocation to type of relationship was randomized, but balanced by the software.

### Results

Table 2 summarizes the most important findings of Study 2. As expected, the IOS scores are substantially and statistically highly significantly different between the three levels of relationships: closest, friend and acquaintance. The same is true for the We Scale, Oneness, and the SCI. (For an illustration of the distribution of the scores see S1 Fig). The scales are also highly correlated with one another, in particular, if we look at all data, but also for all subgroups.

**Table 2:** Mean scores and intercorrelations of scales in Study 2.

A: Mean Scores								
	IOS		We		Oneness		SCI	
	M	SD	M	SD	M	SD	M	SD
**Close**	5.2	1.2	5.6	1.6	5.4	1.3	12.3	2.2
**Friend**	3.7	1.3	4.3	1.5	4.0	1.2	9.4	2.4
**Acquaintance**	2.3	1.3	2.6	1.7	2.5	1.4	5.2	3.1
**Kruskal-Wallisχ** ^**2**^ **(2)**	56.4***		41.9***		55.3***		66.6***	
**B: Intercorrelations**								
			**We**				**SCI**	
**IOS all**			0.79**				0.83**	
**IOS closest**			0.66**				0.41**	
**IOS friend**			0.52**				0.70**	
**IOS acquaintance**			0.73**				0.80**	
**We all**							0.75**	
**We closest**							0.36*	
**We friend**							0.56**	
**We acquaintance**							0.72**	
**Oneness all**							0.84**	
**Oneness closest**							0.42**	
**Oneness friend**							0.71**	
**Oneness acquaint.**							0.81**	

Panel A: mean scores; Panel B: intercorrelations. IOS, We Scale and Oneness are between 1 and 7; SCI is between 2 and 14 (higher scores indicate higher closeness). Oneness is the average of IOS and We Scale. Number of observations: Close (n = 41); Friend (n = 37); Acquaintance (n = 42).

* p < 0.1

** p < 0.05

*** p < 0.01

A further interesting observation is that for close relationships the scores of all four scales of Study 2 are only insignificantly different from the respective scores in the close relationships of Study 1. All p-values exceed 0.32 (rank sum tests). This lends further support to the replicability of the closeness scores we are mainly interested in.

### Discussion

Study 2 confirmed the expectation that the class of relationship—close, friend or acquaintance—has a strong influence on all three measured relationship closeness scores. This observation prepares the ground for the most important part of this paper, Study 3, which is our most comprehensive evaluation of the IOS Scale.

## Study 3

In this study we replicate the IOS, the We and the SCI Scales, and the RCI for each of the three classes of relationships investigated in Study 2. In addition, we add the Personal Acquaintance Measure (PAM) by Starzyk et al. (SHFM, [11]), Rubin's Liking and Loving Scales [13], as well as the Balanced Inventory of Desirable Reporting (BIDR) by Paulhus and Reid [26] and a short personality questionnaire [27].

While the RCI is mostly seen as measuring close relationships, PAM intends to broaden the measurement to "acquaintance", which is more general than our usage of that term so far. SHFM use the word acquaintance "to refer to the degree to which one is familiar with or has knowledge about another person" (SHFM [11], p. 833) and the goal is to develop a measure that applies to any relationship. Study 1 in SHFM derives the PAM by comprehensively measuring acquaintance quantity (how much time people spend together) and acquaintance quality (the degree to which people self-disclose). The results suggest that acquaintance is defined by six dimensions: Knowledge of Goals; Frequency of Interactions; Social Network Familiarity; Self-Disclosure; Physical Intimacy; and Duration (see SHFM, Study 1, for the derivation of these dimensions, and SHFM Appendix, p. 847, for the questions that constitute these dimensions).

### Methods

We recruited 452 people on MTurk for this study (SHFM had 297 participants). Participants received a flat payment of £2.50 for participation. Most participants finished the task in about 20 minutes. After accepting the HIT, participants read the same introductory text as in Study 1. Participants were randomly allocated to treatment, that is, the class of relationship closeness (close, friend or acquaintance) we wanted people to focus on. We used the exact same texts and treatments as in our Study 2. Participants then entered the initial of the target person they had in mind and the software automatically inserted this initial wherever necessary. Within a treatment (i.e., class of relationship) the order at which the different questionnaires were presented to subjects was randomized across subjects, except for the BIDR and the personality questionnaire which came at the end for everybody.

Compared to Study 1 and 2, Study 3 has four main new elements: the PAM, the Loving and Liking Scales, the BIDR to control for social desirability bias, and a personality questionnaire. The IOS Scale, the We Scale and the SCI Scale are exactly the same as in Studies 1 and 2, and the RCI Scale is the same as in Study 1, except that the target person is now treatment dependent (that is, people are now, like in Study 2, prompted to identify a closest person, a friend, or an acquaintance). Except for the We Scale and the personality item our Study 3 has the same elements and structure as Study 2 in SHFM.

The original PAM questionnaire consists of 18 questions (documented in the Appendix of SHFM [11], p. 847) of which we administered only 15 questions. We dropped the three questions that make up the dimension Physical Intimacy (items 2, 11, and 16). We deem these questions unimportant for our purposes and they were also not covered by our ethical review. Answers are on a 5-point scale, from "definitely false" to "definitely true". Subscales are created by summing the appropriate items, according to the scoring instructions given by SHFM ([11], Appendix, p. 847). Scores for the individual factors can be between 0 and 12, and the total PAM score can be between 0 and 60. Higher scores indicate higher closeness.

The Liking Scale and the Loving Scale each consist of 13 questions (see [13], Table 1, p. 267). The Liking Scale asks questions about the positive evaluation of the target person, and the Loving Scale assesses the affection for that target person. Answers are on a 9-point scale, from 1 = "not at all true" to 9 = "definitely true". Total scores can therefore be between 13 and 117 for each scale.

The BIDR is a well-known instrument to test for social desirability bias. It consists of 40 items (scored on a 5-point scale from "not true" to "very true") and aims to identify self-deception, and impression management [26]. We also include a short personality questionnaire [27] to see whether any of the Big Five dimensions matter for IOS ratings.

### Results

Since the design of Study 3 includes replications of Study 1 and 2 we start by investigating to what extent we have replicated our previous results with the new and much larger sample of Study 3. Table 3 reports the six pair wise correlations that are also included in Table 2 (IOS Scale, the RCI Total, RCI Frequency, RCI Diversity, RCI Strength and SCI). In addition, Table 3 also reports the pair wise correlations with the most important variables new to Study 3—the Loving and the Liking Scale, and the PAM Scale.

**Table 3:** Correlations among IOS Scale, RCI Scales, SCI Scale, Love and Liking Scales, and PAM Scale in Study 3.

Scale	1	2	3	4	5	6	7	8	9
**1. IOS Scale**		.67	.49	.55	.66	.82	.79	.56	.70
**2. RCI Total**			.88	.88	.82	.67	.75	.47	.64
**3. RCI Frequency**				.73	.53	.48	.55	.34	.46
**4. RCI Diversity**					.60	.58	.65	.43	.60
**5. RCI Strength**						.67	.76	.44	.62
**6. SCI Scale**							.86	.62	.80
**7. Loving Scale**								.70	.77
**8. Liking Scale**									.57
**9. PAM Scale**									
**Mean**	4.11	11.67	4.12	3.57	3.98	9.61	69.86	84.96	41.67
**SD**	1.82	5.72	2.47	1.85	2.3	3.75	27.61	21.34	11.23
**Obs. No.**	450	436	452	452	436	452	451	450	446

All correlations are significant at p<.01.

The replication results are very encouraging. All correlations have the expected sign. However, correlations tend to be stronger in our larger but also more diverse sample that now not only includes close relationships (as in Study 1) but also relationships with friends and acquaintances. The correlations of the IOS Scale with the other scales are also all highly significant if we look at them for each of the three classes of relationship (close, friends, acquaintances) separately. Thus, the results from Table 3 are not an artifact of aggregation. A further piece of evidence before we come to the main finding is that the results from Study 2 are replicated closely (S4 Table and S2 Fig).

We now turn to the main findings of Study 3, which concern the PAM Scale, the Liking and Loving Scales, and the RCI Scale across the three classes of relationship closeness: close, friends and acquaintances. The PAM Scale, and the Liking and Loving Scales, and the RCI Scale and it subscales differ highly significantly between the three classes of relationships: for PAM, χ^2^(2) = 223.0, p = 0.0001; Liking Scale: χ^2^(2) = 99.3, p = 0.0001; Loving Scale: χ^2^(2) = 241.1, p = 0.0001; RCI: χ^2^(2) = 174.8, p = 0.0001 (Kruskal-Wallis tests). All five factors of PAM are also highly significantly (p = 0.0001, Kruskal Wallis tests) different between classes of relationships (S5 Table).

Table 4 reports the results of our study in comparison with the findings of SHFM. The top part of Table 4 records our findings and, for ease of comparison, the bottom part includes the results of SHFM [11] (their Study 2, Table 5). The table reports the correlations of the relationship inventories (IOS Scale, RCI Scales, Loving and Liking Scales) as well as the BIDR variables with the five factors of PAM. The final column reports the correlations with the total PAM scores.

**Table 4:** Personal Acquaintance Measure (PAM) correlations.

*Our Study*						
	D	FOI	KOG	SD	SNF	Total
**Relationship inventories:**						
**Inclusion of the Other in the Self Scale**	0.50**	0.49**	0.61**	0.28**	0.54**	0.70**
**RCI Total**	0.33**	0.67**	0.57**	0.17**	0.46**	0.64**
**RCI Frequency**	0.19**	0.55**	0.44**	0.10*	0.28**	0.46**
**RCI Diversity**	0.29**	0.62**	0.51**	0.19**	0.44**	0.60**
**RCI Strength**	0.38**	0.56**	0.54**	0.15**	0.48**	0.62**
**Love scale**	0.54**	0.53**	0.70**	0.32**	0.59**	0.77**
**Liking scale**	0.34**	0.37**	0.58**	0.35**	0.40**	0.57**
**Social desirable responding:**						
**BIDR Total**	0.13**	0.12*	0.26**	0.25**	0.15**	0.26**
**BIDR Self-Deceptive Enhancement**	0.15**	0.17**	0.26**	0.20**	0.18**	0.27**
**BIDR Impression management**	0.09	0.03	0.19**	0.24**	0.08	0.17**
***SHFM [11]—Study 2, Table 5***						
	**D**	**FOI**	**KOG**	**SD**	**SNF**	**Total**
**Relationship inventories:**						
**Inclusion of the Other in the Self Scale**	0.57**	0.25**	0.65**	0.57**	0.54**	0.76**
**RCI Total**	0.16**	0.47**	0.45**	0.36**	0.38**	0.54**
**RCI Frequency**	-0.04	0.46**	0.23**	0.21**	0.21**	0.31**
**RCI Diversity**	-0.02	0.47**	0.31**	0.23**	0.30**	0.37**
**RCI strength**	0.56**	0.15**	0.61**	0.48**	0.46**	0.69**
**Loving scale**	0.69**	0.13*	0.76**	0.63**	0.58**	0.84**
**Liking scale**	0.49**	0.16**	0.64**	0.53**	0.45**	0.64**
**Social desirable responding:**						
**BIDR Total**	0.12**	0.02	0.15*	0.16**	0.05	0.15*
**BIDR Self-Deceptive Enhancement**	0.11	0.1	0.20**	0.15*	0.12*	0.22**
**BIDR Impression management**	0.09	-0.05	0.07	0.13*	-0.01	0.05

The top part reports our Study 3 results and the bottom part the results of Study 2 (Table 5) of SHFM [11]**.** D = Duration; FOI = Frequency of Interaction; KOG = Knowledge of Goals; SD = Self-Disclosure; SNF = Social Network Familiarity; RCI = Relationship Closeness Inventory; BIDR = Balanced inventory of Desirable Responding.

** p < 0.01

* p < 0.05.

**Table 5:** Explaining IOS scores in Study 3 with socio-demographics, IRC, and Personality.

	All	Close	Friend	Acquaintance
**Female myself**	-0.2343	-0.0998	-0.2110	-0.3733
	(0.1626)	(0.3225)	(0.2912)	(0.2830)
**Female other**	-0.0459	0.1493	-0.1007	-0.1778
	(0.1634)	(0.3217)	(0.3072)	(0.3037)
**Both female**	0.0944	-0.4002	0.1047	0.3719
	(0.2540)	(0.4672)	(0.4140)	(0.4268)
**Age**	-0.0048	-0.0140	-0.0021	-0.0087
	(0.0052)	(0.0101)	(0.0090)	(0.0088)
**Time known**	0.0135**	0.0157	0.0140	0.0171
	(0.0062)	(0.0095)	(0.0087)	(0.0242)
**Friend**	-0.2896			
	(0.2620)			
**Acquaintance**	-0.1583			
	(0.2478)			
**IRC**	0.7658*	0.7853**	0.7658**	0.7667**
	(0.0552)	(0.0960)	(0.0998)	(0.0806)
**Extraversion**	0.0160	-0.0219	0.0371	0.0283
	(0.0152)	(0.0288)	(0.0250)	(0.0295)
**Agreeableness**	0.0268	0.0777	-0.0104	0.0443
	(0.0216)	(0.0425)	(0.0312)	(0.0453)
**Conscientiousness**	-0.0425	-0.0729*	-0.0161	-0.0594
	(0.0237)	(0.0354)	(0.0521)	(0.0390)
**Stability**	0.0078	0.0390	-0.0204	0.0003
	(0.0190)	(0.0391)	(0.0336)	(0.0273)
**Openness**	-0.0551	0.0105	-0.0852*	-0.0701
	(0.0242)	(0.0499)	(0.0413)	(0.0422)
**N**	418	137	139	142
**Χ** ^**2**^	352.1	89.9	78.1	113.0
**Pseudo R** ^**2**^	.3270	.2001	.1851	.2676

Female myself and Female target are dummies (1 if female) of the subject and their target individual, respectively. Both female is an interaction variable of Female myself*Female target. Age is biological years of the subject. Time known refers to the years the subject had known the target person. Friend and Acquaintance are dummies of the respective treatment (with Close being the omitted reference group). IRC is our Index of Relationship Closeness. The Big Five are taken from [27]. The regression is ordered probit.

** p < 0.01

* p < 0.05.

Our findings are largely in line with the results of SHFM. This holds for the five factors of the PAM we look at and in particular the total score of the PAM (last column), where all correlations are at least 0.46 (in SHFM the respective correlations are at least 0.31). In our data, BIDR variables that control for social desirability bias in responding are a bit more strongly correlated with the respective PAM variables than in SHFM. However, as in SHFM, all correlations are substantially smaller than for the relationship inventories. SHFM argue that their results show discriminant and convergent validity. Our findings broadly replicate their results and therefore support this conclusion.

### Discussion

In Study 3, we have successfully replicated the PAM Scale and also the findings from our Studies 1 and 2. The most important outcome for our purposes is that the IOS Scale is highly significantly correlated with conceptually different measures of relationship closeness: the RCI Scale, the Liking and Loving Scales, and the PAM Scale. The most important finding in this respect is in Table 3, which shows that the IOS Scale is highly correlated with all scales intended to measure the closeness of relationships. Based on this fact, as well as high intercorrelations between the various scales, we will attempt in the next section to construct an "Index of Relationship Closeness" (IRC) and then check how it correlates with the IOS Scale.

## IOS as a Compact Measure of the Closeness of Relationships

The fact that the RCI Scale, the SCI Scale, the We Scale, the Loving and Liking Scales, and the PAM Scale are highly correlated variables (Table 3) suggests that these scales measure the same latent construct, despite having different conceptual foundations [1, 11–13, 22]. Our goal is to utilize this fact of strong positive correlations by constructing an Index of Relationship Closeness (IRC) and to see how IRC correlates with the IOS Scale. If the IOS Scale is highly correlated with IRC as we do expect from our three studies, then this would suggest that the IOS Scale is a very convenient and psychologically meaningful tool for measuring relationship closeness. A strong correlation would therefore vindicate AAS's claim that the IOS Scale captures "something in the respondent's perception of a relationship that is consistent with many theoretical orientations" (AAS [1], p. 598).

**Fig 2:**
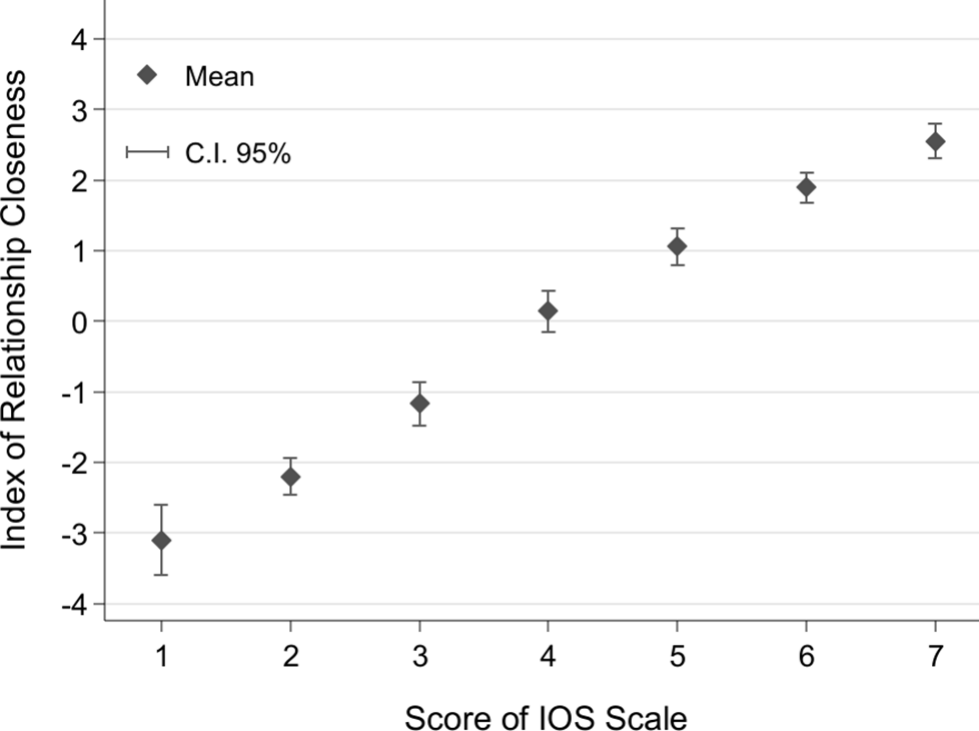
The link between the Index of Relationship Closeness and IOS. ‘The Index of Relationship Closeness’ is the principal component of five measurements of the closeness of relationships: RCI and SCI [12], We Scale [22], Loving and Liking Scale [13], and PAM Scale [11].

In order to construct an IRC, we perform a Principal Component Analysis (PCA) to reduce the dimensionality of our data set by identifying the components which explain a significant fraction of the variance across the above six variables of interest. The first component has an eigenvalue of 4.50, accounting for 75% of the variance. The second biggest eigenvalue is 0.55 accounting for 9% of the variance. In accordance with the Kaiser criterion (which drops any component with eigenvalues smaller than 1) and the Scree test (involving a plot of the eigenvalues in decreasing order of their magnitude against the component numbers to determine where the eigenvalues level off—see S2 Text), we confidently retain only the first component. For this component, we obtained composite scores for each individual in our data set. These scores constitute our IRC measure. In our data set, IRC ranges from -5.02 to 3.70 and has a mean of zero and a standard deviation equal to 2.12. Higher values represent closer relationships. Fig 2 plots IRC against each IOS score value, by reporting the relevant means and confidence intervals (at the 95% level).

The relation between IRC and IOS appears to be almost linear, with a slope of almost one. The Spearman rank correlation between IRC and the IOS Scale is ρ = 0.85 (p<0.0001). The highly significantly positive correlation between IRC and the IOS Scale not only holds for all classes of relationships pooled (as shown in Fig 2), but also separately for each class of relationship: Spearman's ρ>.64, p<.00001 (regressions show similar results). We see these results as a strong endorsement of the usefulness of the IOS task.

An extension of the IOS Scale is the Oneness Scale, which combines the IOS Scale with the We Scale [22]. If we run the PCA excluding the We Scale and then regress the resulting IRC scores on the Oneness scores, we get a *β* coefficient equal to 0.929 (p<0.0001). If we regress those IRC scores on the IOS scores, we get a *β* coefficient of 0.870 (p<0.0001). Thus, the Oneness Scale does even slightly better than the IOS Scale alone.

Finally, we use regression analysis to see to what extent socio-demographics, duration of knowledge of the other person, and personality factors [27] matter for the IOS ratings. We estimate ordered probit models with the IOS score as the dependent variable. Table 5 shows four estimations: one with the data pooled for all classes of relationships, and three estimations for each class of relationship.

The most important result of the pooled data set is that IRC remains highly significant, with a z-score exceeding 13. Own and other gender and their interaction do not matter, and neither does age. The time the target person is known to the subject increases the IOS score slightly and significantly (p = .030). Of all the personality measures, only Openness matters slightly negatively (at p = .023).

The remaining three models regress the IOS score on the same set of variables but separately for each class of relationship. The most important result is that IRC remains highly significant for all classes of relationships. That is, the IOS score is also a good predictor of the closeness of relationships *within* a class of relationships, and not just between relationship classes. This is comforting because IOS, as well as all relationship scales, vary substantially within each level of relationship (S1 and S2 Figs).

## Conclusion

In this paper we presented three studies with the goal of investigating the usefulness of the IOS Scale [1] for measuring relationship closeness. We conducted three studies by using comprehensive relationship inventories to test their correlations with IOS: the Relationship Closeness Inventory, the related Social Closeness Index [12] and the "We Scale" [22] (Studies 1 to 3), the Liking and Loving Scales [13] (Study 3) and the Personal Acquaintance Measure [11] (Study 3). All studies were successful in the sense that the originally reported results also hold in similar magnitude, for a non-student diverse adult population recruited via an online platform (MTurk). The results are also a validation of the use of MTurk in this context because our re-evaluations of the various relationship measures cohere very closely with results from the corresponding studies run using paper and pencil technology in the classroom and with undergraduates as participants.

Most importantly for our purposes, the IOS Scale is highly significantly positively correlated with a new Index of Relationship Closeness (IRC), which we derived from the various relationship inventories we scrutinized across our three studies. Our overall conclusion, therefore, is that the IOS Scale is not only extremely easy to use but it is also highly replicable and psychologically meaningful from various perspectives of relationship closeness. It therefore recommends itself as a handy tool to measure perceived relationship closeness without the need of administering detailed inventories to achieve that goal.

## Supporting Information

S1 Dataset The raw data of our three studies in Excel format. (XLSX)

S1 Fig Distribution of scores of the IOS Scale, the We Scale and the SCI Scale by type of relationship. Number of observations: Close (n = 41); Friend (n = 37); Acquaintance (n = 42). For summary statistics and Kruskal-Wallis test results see Table 2 in the main text.
(PDF)

S2 Fig Distribution of scores of the IOS Scale, the We Scale, the SCI Scale, the RCI, the Loving & Liking Scale (pooled), and the PAM Scale by type of relationship in Study 3. Number of observations: n = 150 for each relationship level. Kruskal-Wallis tests show that for all scales, scores are distributed highly significantly differently across different levels of relationship closeness (χ^2^(2) > 173.99, p< .0005).
(PDF)

S1 Table Cronbach's alpha for the Relationship Closeness Inventory (RCI) and its subscales. BSO refers to Berscheid et al. [12] and AAS to Aron et al. [1]. AAS do not report an alpha for the RCI Diversity scale.
(DOCX)

S2 Table Intercorrelations of RCI subscale scores of closest relationships by relationship type and gender of respondent. The data from BSO are taken from their Table 1 ([12], p. 797).
(DOCX)

S3 Table RCI scores by relationship type and RCI subscale, for our data and BSO. BSO data are taken from [12], Table 2. The subscales are between 1 and 10, and the Total scale is between 3 and 30.
(DOCX)

S4 Table Mean scores (panel A) and intercorrelations of scales (panel B). IOS, We Scale and Oneness are between 1 and 7; SCI is between 2 and 14 (higher scores indicate higher closeness). Oneness is the average of IOS and We Scale. Subgroup correlations are with subgroup of respective scale. ** p < 0.05; *** p < 0.01.
(DOCX)

S5 Table Mean scores of PAM factors and the Loving and Liking Scale for level of acquaintance. PAM subscales are between 0 and 12; PAM Total is between 0 and 60. The Loving and Liking Scale are each between 13 and 117. The RCI subscales are between 1 and 10, and the RCI Total is between 3 and 30.
(DOCX)

S1 Text We document the full set of questions used for Study 3. The questionnaires of Studies 1 and 2 only used a subset.
(DOCX)

S2 Text Performed using the pca command in Stata 13. (DOCX)
