# Supplementary material for: Measuring the Closeness of Relationships: A Comprehensive Evaluation of the 'Inclusion of the Other in the Self' Scale
Source: PLoS One. 2015 Jun 12;10(6):e0129478. doi: 10.1371/journal.pone.0129478 (PMC4466912; doi:10.1371/journal.pone.0129478)
Supplement: S1 Fig — Number of observations: Close (n = 41); Friend (n = 37); Acquaintance (n = 42). For summary statistics and Kruskal-Wallis test results see Table 2 in the main text. (PDF) [file pone.0129478.s002.pdf]

S1 Figure.

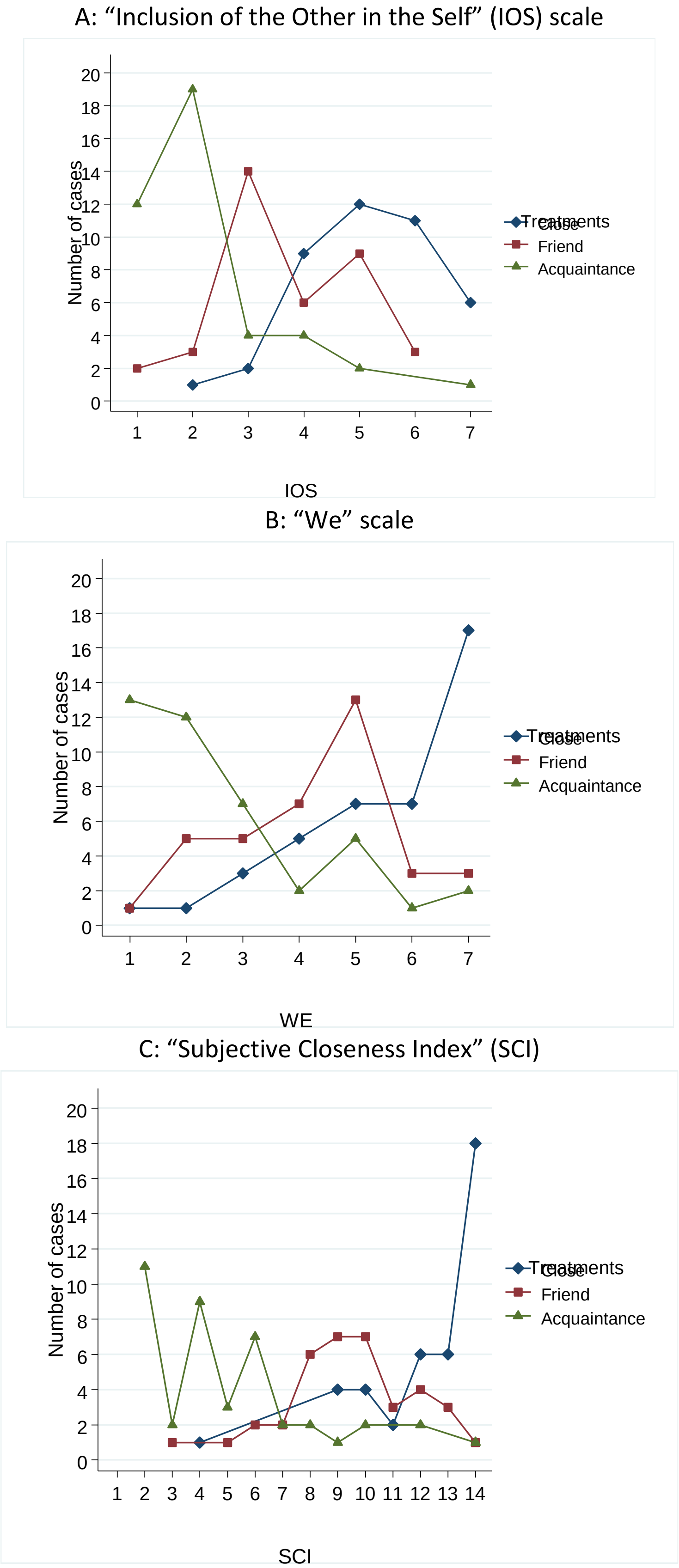
A: “Inclusion of the Other in the Self” (IOS) scale
Number of cases
IOS
Treatments
Close
Friend
Acquaintance
B: “We” scale
Number of cases
WE
Treatments
Close
Friend
Acquaintance
C: “Subjective Closeness Index” (SCI)
Number of cases
SCI
Treatments
Close
Friend
Acquaintance
