# Supplementary material for: Measuring the Closeness of Relationships: A Comprehensive Evaluation of the 'Inclusion of the Other in the Self' Scale
Source: PLoS One. 2015 Jun 12;10(6):e0129478. doi: 10.1371/journal.pone.0129478 (PMC4466912; doi:10.1371/journal.pone.0129478)

S2 Figure.

A: “Inclusion of the Other in the Self” (IOS) scale

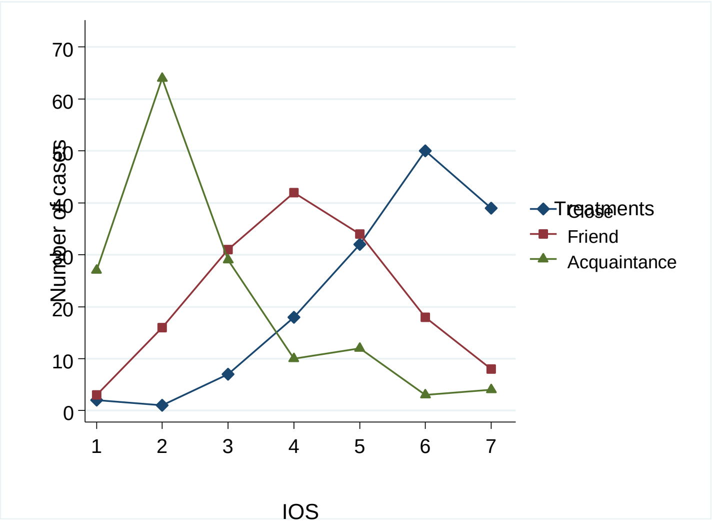

B: “We” scale

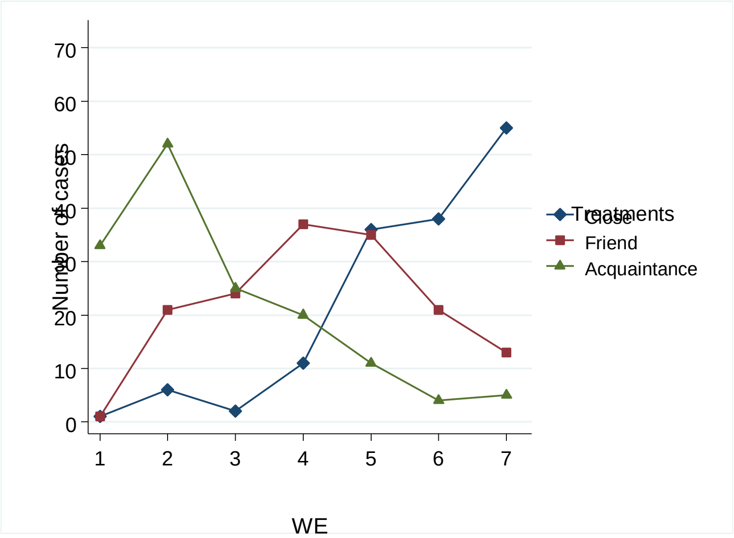

C: “Subjective Closeness Index” (SCI)

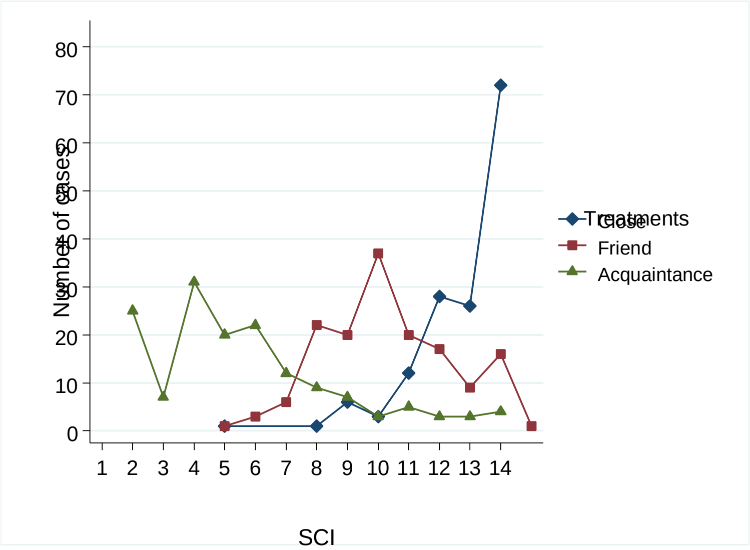

D: “Relationship Closeness Inventory” (RCI)

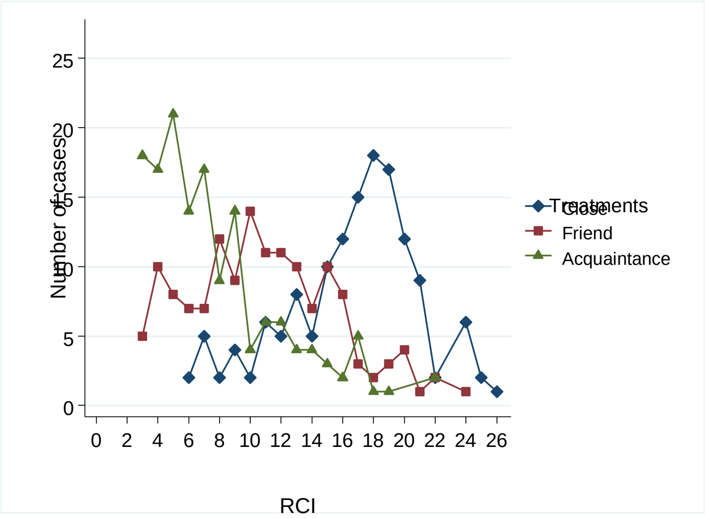

E: “Love and Liking” Scales

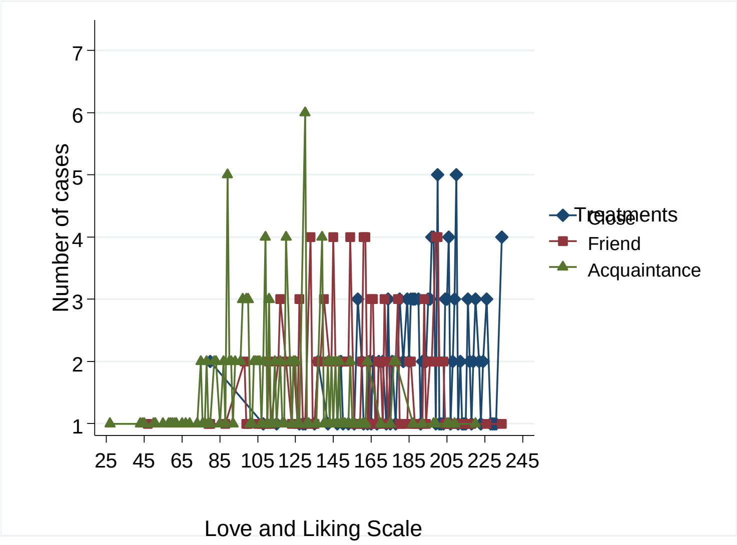

F: “Personal Acquaintance Measure” (PAM)

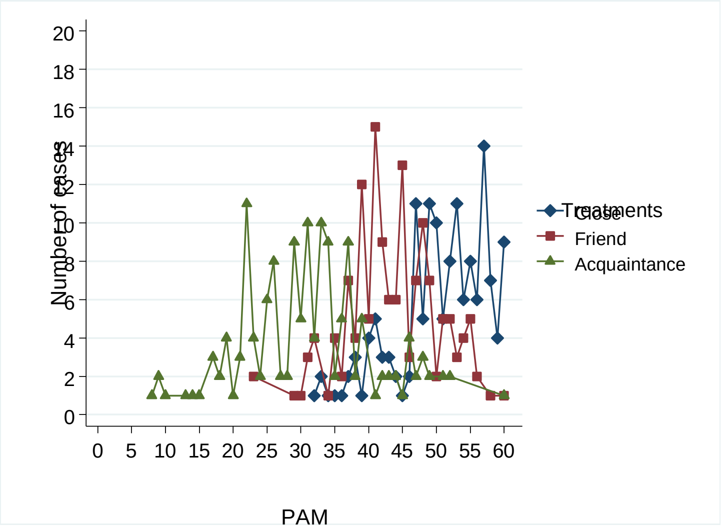

Supplement: S2 Fig — Number of observations: n = 150 for each relationship level. Kruskal-Wallis tests show that for all scales, scores are distributed highly significantly differently across different levels of relationship closeness (χ2(2) > 173.99, p< .0005). (PDF) [file pone.0129478.s003.pdf]
