# Supplementary material for: Measuring the Closeness of Relationships: A Comprehensive Evaluation of the 'Inclusion of the Other in the Self' Scale
Source: PLoS One. 2015 Jun 12;10(6):e0129478. doi: 10.1371/journal.pone.0129478 (PMC4466912; doi:10.1371/journal.pone.0129478)
Supplement: S1 Table — BSO refers to Berscheid et al. [12] and AAS to Aron et al. [1]. AAS do not report an alpha for the RCI Diversity scale. (DOCX) [file pone.0129478.s004.docx]

**S1 Table.**

|  | **Our sample** | **BSO** | **AAS** |
| --- | --- | --- | --- |
| **RCI Total** | .64 | .62 | .66 |
| **RCI Frequency** | .65 | .56 | .85 |
| **RCI Diversity** | .81 | .87 | - |
| **RCI Strength** | .94 | .90 | .88 |
