# Supplementary material for: Measuring the Closeness of Relationships: A Comprehensive Evaluation of the 'Inclusion of the Other in the Self' Scale
Source: PLoS One. 2015 Jun 12;10(6):e0129478. doi: 10.1371/journal.pone.0129478 (PMC4466912; doi:10.1371/journal.pone.0129478)
Supplement: S2 Table — The data from BSO are taken from their Table 1 ([12], p. 797). (DOCX) [file pone.0129478.s005.docx]

**S2 Table.**

|  | **Our study** | | **BSO** | | |
| --- | --- | --- | --- | --- | --- |
|  | **Diversity** | **Strength** | **Diversity** | **Strength** | |
| **All relationship types** (N=200, 241) | | | | | |
| **Frequency** | .51** | .36** | .44* | .30* | |
| **Diversity** | - | .30** | - | .31* | |
| **Romantic relationships** (N=136, 114) | | | | | |
| **Frequency** | .33** | .25** | .34** | .30** | |
| **Diversity** | - | .07 | - | .39** | |
| **Friend relationships** (N=33, 87) | | | | | |
| **Frequency** | .64** | .12 | .49** | .27** | |
| **Diversity** | - | .25 | - | .20 | |
| **Family relationships** (N=21, 34) | | | | | |
| **Frequency** | .43 | .05 | .45** | .06 | |
| **Diversity** | - | .54* | - | -.05 | |
| **Male respondents** (N=111, 116) |  |  |  |  | |
| **Frequency** | .43** | .45** | .46** | .19** |  |
| **Diversity** | - | .33** | - | .25** |  |
| **Female respondents** (N=86, 125) | | | | |  |
| **Frequency** | .59** | .24* | .42** | .41** |  |
| **Diversity** | - | .30** | - | .39** |  |
