# Supplementary material for: Measuring the Closeness of Relationships: A Comprehensive Evaluation of the 'Inclusion of the Other in the Self' Scale
Source: PLoS One. 2015 Jun 12;10(6):e0129478. doi: 10.1371/journal.pone.0129478 (PMC4466912; doi:10.1371/journal.pone.0129478)
Supplement: S3 Table — BSO data are taken from [12], Table 2. The subscales are between 1 and 10, and the Total scale is between 3 and 30. (DOCX) [file pone.0129478.s006.docx]

**S3 Table.**

|  | **Relationship** **type** | | | | | | | |
| --- | --- | --- | --- | --- | --- | --- | --- | --- |
|  | **All** | | **Romantic** | | **Friend** | | **Family** | |
| **RCI measure** | **Mean** | **SD** | **Mean** | **SD** | **Mean** | **SD** | **Mean** | **SD** |
| **A: Our data** | | | | | | | | |
|  | | | | | | | | |
| **Frequency** | 5.72 | 2.16 | 6.29 | 1.83 | 4.15 | 2.18 | 5.05 | 2.01 |
| **Diversity** | 5.07 | 1.59 | 5.46 | 1.40 | 4.42 | 1.58 | 4.29 | 1.31 |
| **Strength** | 5.94 | 1.85 | 6.51 | 1.64 | 4.81 | 1.75 | 4.35 | 1.60 |
| **Total** | 16.80 | 4.26 | 18.25 | 3.40 | 13.59 | 3.99 | 13.75 | 3.68 |
|  |  |  |  |  |  |  |  |  |
| **B: BSO** | | | | | | | | |
|  | | | | | | | | |
| **Frequency** | 3.30 | 2.16 | 3.84 | 2.24 | 2.89 | 2.05 | 2.74 | 1.88 |
| **Diversity** | 3.96 | 1.86 | 4.49 | 2.01 | 3.58 | 1.71 | 3.47 | 1.08 |
| **Strength** | 4.98 | 1.63 | 5.52 | 165 | 4.28 | 1.34 | 4.91 | 1.55 |
| **Total** | 12.24 | 4.29 | 13.85 | 4.44 | 10.74 | 3.84 | 11.12 | 3.02 |
