# Supplementary material for: Measuring the Closeness of Relationships: A Comprehensive Evaluation of the 'Inclusion of the Other in the Self' Scale
Source: PLoS One. 2015 Jun 12;10(6):e0129478. doi: 10.1371/journal.pone.0129478 (PMC4466912; doi:10.1371/journal.pone.0129478)
Supplement: S4 Table — IOS, We Scale and Oneness are between 1 and 7; SCI is between 2 and 14 (higher scores indicate higher closeness). Oneness is the average of IOS and We Scale. Subgroup correlations are with subgroup of respective scale. ** p < 0.05; *** p < 0.01. (DOCX) [file pone.0129478.s007.docx]

**S4 Table.**

|  | **A: Mean Scores** | | | | | | | | | | | | | | |  |
| --- | --- | --- | --- | --- | --- | --- | --- | --- | --- | --- | --- | --- | --- | --- | --- | --- |
|  | **IOS** | | | **We** | | | | **Oneness** | | | | | **SCI** | | | |
|  | **M** | **SD** | | **M** | | **SD** | | **M** | | **SD** | | | **M** | **SD** | | |
| **Close** (n=149) | 5.57 | 1.29 | | 5.74 | | 1.34 | | 5.66 | | | 1.21 | | 12.83 | | 1.55 | |
| **Friend** (n=152) | 4.14 | 1.40 | | 4.31 | | 1.50 | | 4.23 | | | 1.34 | | 10.34 | | 2.10 | |
| **Acquaintance** (n=150) | 2.60 | 1.42 | | 2.71 | | 1.54 | | 2.64 | | | 1.39 | | 5.71 | | 3.04 | |
| Kruskal-Wallis χ^2^(2) with ties | 198.59** | | | 188.79** | | | | 209.31** | | | | | 268.21** | | | |
|  |  | | | | | | | | | | | | | | | |
|  | **B: Intercorrelations** | | | | | | | | | | | | | | | |
|  | **We** | | | |  | |  | |  | | | **SCI** | | | | |
|  |  | |  | |  | |  | |  | | |  | | | | |
| **IOS all** | 0.85** | | | |  | |  | |  | | | 0.82** | | | | |
| **IOS close** | 0.70** | | | |  | |  | |  | | | 0.51** | | | | |
| **IOS friend** | 0.71** | | | |  | |  | |  | | | 0.64** | | | | |
| **IOS acquaintance** | 0.81** | | | |  | |  | |  | | | 0.76** | | | | |
| **We all** |  |  |  | |  | |  | |  | | | 0.79** | | | | |
| **We closest** |  |  |  | |  | |  | |  | | | 0.48** | | | | |
| **We friend** |  |  |  | |  | |  | |  | | | 0.66** | | | | |
| **We acquaintance** |  |  |  | |  | |  | |  | | | 0.66** | | | | |
| **Oneness all** |  |  |  | |  | |  | |  | | | 0.84** | | | | |
| **Oneness close** |  |  |  | |  | |  | |  | | | 0.54** | | | | |
| **Oneness friend** |  |  |  | |  | |  | |  | | | 0.71** | | | | |
| **Oneness acquaintance** |  |  |  | |  | |  | |  | | | 0.77** | | | | |
|  |  |  |  | |  | |  | |  | | |  | | | | |
