# Supplementary material for: Measuring the Closeness of Relationships: A Comprehensive Evaluation of the 'Inclusion of the Other in the Self' Scale
Source: PLoS One. 2015 Jun 12;10(6):e0129478. doi: 10.1371/journal.pone.0129478 (PMC4466912; doi:10.1371/journal.pone.0129478)
Supplement: S5 Table — PAM subscales are between 0 and 12; PAM Total is between 0 and 60. The Loving and Liking Scale are each between 13 and 117. The RCI subscales are between 1 and 10, and the RCI Total is between 3 and 30. (DOCX) [file pone.0129478.s008.docx]

**S5 Table.**

|  | **Close** |  | **Friend** |  | **Acquaintance** | |  |
| --- | --- | --- | --- | --- | --- | --- | --- |
|  | **M** | **SD** | **M** | **SD** | **M** | **SD** | **Chi2** |
| **PAM Duration** | 9.95 | 2.60 | 9.23 | 3.04 | 5.15 | 3.75 | 131.89 |
| **PAM Frequency** | 10.41 | 2.46 | 6.81 | 3.43 | 6.41 | 3.63 | 123.89 |
| **PAM Knowledge** | 10.74 | 1.59 | 9.59 | 1.88 | 6.73 | 2.84 | 167.26 |
| **Pam Self-Disclosure** | 8.96 | 2.72 | 8.36 | 2.45 | 6.86 | 2.58 | 51.37 |
| **PAM Network Fam.** | 10.33 | 2.04 | 9.61 | 2.07 | 5.96 | 3.48 | 146.80 |
| **PAM Total** | 50.37 | 6.86 | 43.57 | 6.86 | 31.16 | 9.77 | 223.02 |
|  |  |  |  |  |  |  |  |
| **Love Scale** | 93.91 | 16.34 | 71.64 | 18.92 | 44.34 | 20.78 | 241.06 |
| **Liking Scale** | 95.66 | 15.76 | 87.63 | 18.77 | 71.81 | 21.77 | 99.27 |
|  |  |  |  |  |  |  |  |
| **RCI Frequency** | 5.52 | 2.16 | 3.86 | 2.39 | 3.00 | 2.17 | 86.65 |
| **RCI Diversity** | 4.97 | 1.54 | 3.35 | 1.68 | 2.40 | 1.33 | 150.29 |
| **RCI Strength** | 5.97 | 1.89 | 3.63 | 1.71 | 2.41 | 1.73 | 186.27 |
| **RCI Total** | 16.48 | 4.34 | 10.86 | 4.75 | 7.82 | 4.28 | 171.80 |
