# Supplementary material for: Measuring the Closeness of Relationships: A Comprehensive Evaluation of the 'Inclusion of the Other in the Self' Scale
Source: PLoS One. 2015 Jun 12;10(6):e0129478. doi: 10.1371/journal.pone.0129478 (PMC4466912; doi:10.1371/journal.pone.0129478)
Supplement: S1 Text — The questionnaires of Studies 1 and 2 only used a subset. (DOCX) [file pone.0129478.s009.docx]

**S1 Text**

**Documentation of the Questionnaires used**

Here, we report the full set of questions we used in Study 3 (for close relationships; note that only the introductory text preceding Q1 was changed depending on the implemented treatment – see main article for implementation details). The questionnaire was implemented using the survey software Qualtrics (<www.qualtrics.com>).

Study 1 and 2 use different subsets of the questions implemented in Study 3:

- Study 1 used Q1-Q10 and Q26-Q64.
- Study 2 used Q1-Q10.

The questions relate to different scales and scholarly articles. That is:

- Q1-Q6 and Q141-Q142 are introductory/background questions;
- Q7 refers to the “Inclusion of the Other in the Self” (‘IOS’ for short) scale proposed by Aron et al. (J Pers Soc Psych 63, 596-612 [1992]);
- Q8 refers to the “We” scale as used in Cialdini et al. (J Pers Soc Psych 73, 481-494 [1997]);
- Q9 and Q10 refer to the “Subjective Closeness Index” (‘SCI’ for short) following Berscheid et al. (J Pers Soc Psych 57, 792-807 [1989]);
- Q11-Q25 refer to the “Personal Acquaintance Measure” (‘PAM’ for short) introduced by Starzyk et al. (J Pers Soc Psych 90, 833-847 [2006]);
- Q26-Q64 refer to the “Relationship Closeness Inventory” (‘RCI’ for short) by Berscheid et al. (J Pers Soc Psych 57, 792-807 [1989]);
- Q65-Q77 and Q78-Q90 refer respectively to Loving and Liking scales by Rubin (J Pers Soc Psych 16, 265-273 [1970]);
- Q91-Q130 refer to the “Balanced Inventory of Desirable Reporting” (‘BIDR’ for short) by Paulhus and Reid (J Pers Soc Psych 60, 307-317 [1991]);
- Q131-Q140 refer to the “Ten-Item Personality Inventory by Gosling et al. (J Res Pers 37, 504-528 [2003]).

**Legend**

[...] The text in square brackets was not displayed to participants;

{X} Placeholder for initial of the first name of the person a participant had in mind;

___ Text entry option;

▼ Drill down choice menu;

- Multiple choice menu, with single answer;
- Multiple choice menu, with multiple answers.

******

Thank you for participating in our HIT! In this HIT we will ask you to respond to a questionnaire on the nature of interpersonal relationships. Our interest is entirely scientific. All answers will be treated confidentially and will only be reported in aggregated statistical form. There are no right or wrong answers in this survey, we are only interested in your honest assessment. If you feel uncomfortable answering some questions you will have opportunities to select “prefer not to answer” as an answer.

We are currently investigating the nature of interpersonal relationships. As part of this study, we would like you to answer the following questions about your relationship with another person. Specifically, we would like you to choose the one person with whom you have the closest, deepest, most involved, and most intimate relationship, and answer the following questions with regard to this particular person. For some of you, this person may be a dating partner or someone with whom you have a romantic relationship. For others of you, this person may be a close, personal friend, family member, or companion. It makes no difference exactly who this person is as long as she or he is the one person with whom you have the closest, deepest, most involved, and most intimate relationship. Please select this person carefully since this decision will affect the rest of this study. With this person in mind, please respond to the following questions.

[Q1] Who is this person? Please give the initial of the first name only.

____________________

[Page break here]

[Q2] What is your gender?

- Male
- Female
- prefer not to say

[Q3] What is {X}'s gender?

- Male
- Female
- prefer not to say

[Q4] What is your age?

____________________

[Q5] How long have you known {X}? Please indicate the number of years and months.

▼ Years

▼ Months

[Page break here]

[Q6] Which of the following best describes your relationship with {X}? (Check only one)

- WORK: co-worker
- WORK: your boss/supervisor
- WORK: your subordinate
- FAMILY: aunt/uncle
- FAMILY: sister/brother
- FAMILY: parent
- FAMILY: cousin
- ROMANTIC: married
- ROMANTIC: engaged
- ROMANTIC: living together
- ROMANTIC: dating only this person
- ROMANTIC: dating this person and others
- FRIEND: close friend (non-romantic)
- FRIEND: casual friend
- ACQUAINTANCE (please give short description) ____________________
- OTHER (please give short description) ____________________
- prefer not to say

[Q7] In the following figure we ask you to consider which of these pairs of circles best describes your relationship with this person (referred to as {X} in all questions that follow). In the figure "X" serves as a placeholder for {X}, that is, you should think of "X" being {X}. By selecting the appropriate number please indicate to what extent you and {X} are connected.

- 1
- 2
- 3
- 4
- 5
- 6
- 7
- prefer not to answer

[Q8] Please, select the appropriate number below to indicate to what extent you would use the term “WE” to characterize you and {X}.

- 1 - Not at all
- 2
- 3
- 4
- 5
- 6
- 7 - very much so
- prefer not to answer

[Page break here]

[Q9] Relative to all your other relationships (both same and opposite sex) how would you characterize your relationship with {X}?

- 1 - not close at all
- 2
- 3
- 4
- 5
- 6
- 7 - very close
- prefer not to answer

[Q10] Relative to what you know about other people's close relationships, how would you characterize your relationship with {X}?

- 1 - not close at all
- 2
- 3
- 4
- 5
- 6
- 7 - very close
- prefer not to answer

Please think of {X}, read each statement carefully, and click the answer that best corresponds to your agreement or disagreement with each statement.

[Q11] I have known {X} for many years.

- Strongly Disagree
- Disagree
- Neither Agree nor Disagree
- Agree
- Strongly Agree
- Prefer Not to Answer

[Q12] I have known {X} for a long time.

- Strongly Disagree
- Disagree
- Neither Agree nor Disagree
- Agree
- Strongly Agree
- Prefer Not to Answer

[Q13] I have gone to parties (social events) with {X}.

- Strongly Disagree
- Disagree
- Neither Agree nor Disagree
- Agree
- Strongly Agree
- Prefer Not to Answer

[Q14] {X} often hides his/her true feelings from me.

- Strongly Disagree
- Disagree
- Neither Agree nor Disagree
- Agree
- Strongly Agree
- Prefer Not to Answer

[Q15] Seeing {X} is part of my weekly routine.

- Strongly Disagree
- Disagree
- Neither Agree nor Disagree
- Agree
- Strongly Agree
- Prefer Not to Answer

[Q16] I know what {X}'s goals are.

- Strongly Disagree
- Disagree
- Neither Agree nor Disagree
- Agree
- Strongly Agree
- Prefer Not to Answer

[Q17] {X} hides his/her true feelings from me.

- Strongly Disagree
- Disagree
- Neither Agree nor Disagree
- Agree
- Strongly Agree
- Prefer Not to Answer

[Q18] {X} has told me about his/her interests.

- Strongly Disagree
- Disagree
- Neither Agree nor Disagree
- Agree
- Strongly Agree
- Prefer Not to Answer

[Q19] I have spent time with {X} and his/her friends.

- Strongly Disagree
- Disagree
- Neither Agree nor Disagree
- Agree
- Strongly Agree
- Prefer Not to Answer

[Q20] {X} avoids showing his/her true feelings around me.

- Strongly Disagree
- Disagree
- Neither Agree nor Disagree
- Agree
- Strongly Agree
- Prefer Not to Answer

[Q21] {X} and I go way back.

- Strongly Disagree
- Disagree
- Neither Agree nor Disagree
- Agree
- Strongly Agree
- Prefer Not to Answer

[Q22] I am familiar with {X}'s friends.

- Strongly Disagree
- Disagree
- Neither Agree nor Disagree
- Agree
- Strongly Agree
- Prefer Not to Answer

[Q23] I see {X} a lot.

- Strongly Disagree
- Disagree
- Neither Agree nor Disagree
- Agree
- Strongly Agree
- Prefer Not to Answer

[Q24] Seeing {X} is part of my daily routine.

- Strongly Disagree
- Disagree
- Neither Agree nor Disagree
- Agree
- Strongly Agree
- Prefer Not to Answer

[Q25] {X} has told me what his/her goals are.

- Strongly Disagree
- Disagree
- Neither Agree nor Disagree
- Agree
- Strongly Agree
- Prefer Not to Answer

We would like you to estimate the amount of time you typically spend alone with {X} during the day. We would like you to make these time estimates by breaking the day into morning, afternoon, and evening, although you should interpret each of these time periods in terms of your own typical daily schedule. (For example, if you work a night shift, "morning" may actually reflect time in the afternoon, but is nevertheless time immediately after waking.) Think back over the past week and select the average amount of time, per day, that you spent alone with {X}, with no one else around, during each time period. If you did not spend any time with {X} in some time periods, select 0 hour(s) and 0 minutes.

[Q26] DURING THE PAST WEEK, what is the average amount of time, per day, that you spent alone with {X} in the MORNING (e.g., between the time you wake and 12 noon)?

▼ Hours

▼ Minutes

[Q27] DURING THE PAST WEEK, what is the average amount of time, per day, that you spent alone with {X} in the AFTERNOON (e.g., between 12 noon and 6pm)?

▼ Hours

▼ Minutes

[Q28] DURING THE PAST WEEK, what is the average amount of time, per day, that you spent alone with {X} in the EVENING (e.g., between 6pm and bedtime)?

▼ Hours

▼ Minutes

[Q29] Compared with the "normal" amount you usually spend alone with {X}, how typical was the past week?

- typical
- not typical

[Q30] The following is a list of different activities that people may engage in over the course of one week. For each of the activities listed, please check all of those that you have engaged in alone with {X} in the past week. Check only those activities that were done alone with {X} and not done with {X} in the presence of others. In the past week, I did the following activities alone with {X} (Check all that apply)

- did laundry
- prepared a meal
- watched TV
- went to an auction/antique show
- attended a non-class lecture or presentation
- went to a restaurant
- went to a grocery store
- went for a walk/drive
- discussed things of a personal nature
- went to a museum/art show
- planned a party/social event
- attended class
- went on a trip (e.g., vacation or weekend)
- cleaned house/apartment
- went to church/religious function
- worked on homework
- spent time together on the internet (e.g., Skype, FaceTime, surfing together, etc)
- discussed things of a non-personal nature
- went to a clothing store
- talked on the phone
- went to a movie
- ate a meal
- participated in a sporting activity
- outdoor recreation (e.g., sailing)
- went to a play
- went to a bar
- visited family
- visited friends
- went to a department, book, hardware store, etc.
- played cards/board game
- attended a sporting event
- exercise (e.g., jogging, aerobics)
- went on an outing (e.g., picnic, beach, zoo, winter carnival)
- wilderness activity (e.g., hunting, hiking, fishing)
- went to a concert
- went dancing
- went to a party
- played music/sang
- other (please describe briefly) ____________________

[Page break here]

The following questions concern the amount of influence {X} has on your thoughts, feelings, and behavior. Using the 7-point scale below, please indicate the extent to which you agree or disagree (from 1 - I strongly disagree to 7 - I strongly agree)

[Q31] {X} will influence my future financial security.

- 1 - I strongly disagree
- 2
- 3
- 4
- 5
- 6
- 7 - I strongly agree
- prefer not to answer

[Q32] {X} does not influence everyday things in my life.

- 1 - I strongly disagree
- 2
- 3
- 4
- 5
- 6
- 7 - I strongly agree
- prefer not to answer

[Q33] {X} influences important things in my life.

- 1 - I strongly disagree
- 2
- 3
- 4
- 5
- 6
- 7 - I strongly agree
- prefer not to answer

[Q34] {X} influences which parties and other social events I attend.

- 1 - I strongly disagree
- 2
- 3
- 4
- 5
- 6
- 7 - I strongly agree
- prefer not to answer

[Q35] {X} influences the extent to which I accept responsibilities in our relationship.

- 1 - I strongly disagree
- 2
- 3
- 4
- 5
- 6
- 7 - I strongly agree
- prefer not to answer

[Q36] {X} does not influence how much time I spend doing household work.

- 1 - I strongly disagree
- 2
- 3
- 4
- 5
- 6
- 7 - I strongly agree
- prefer not to answer

[Q37] {X} does not influence how I choose to spend my money.

- 1 - I strongly disagree
- 2
- 3
- 4
- 5
- 6
- 7 - I strongly agree
- prefer not to answer

[Q38] {X} influences the way I feel about myself.

- 1 - I strongly disagree
- 2
- 3
- 4
- 5
- 6
- 7 - I strongly agree
- prefer not to answer

[Q39] {X} does not influence my moods.

- 1 - I strongly disagree
- 2
- 3
- 4
- 5
- 6
- 7 - I strongly agree
- prefer not to answer

[Q40] {X} influences the basic values that I hold.

- 1 - I strongly disagree
- 2
- 3
- 4
- 5
- 6
- 7 - I strongly agree
- prefer not to answer

[Q41] {X} does not influence the opinions that I have of other important people in my life.

- 1 - I strongly disagree
- 2
- 3
- 4
- 5
- 6
- 7 - I strongly agree
- prefer not to answer

[Q42] {X} does not influence when I see, and the amount of time I spend with, my family.

- 1 - I strongly disagree
- 2
- 3
- 4
- 5
- 6
- 7 - I strongly agree
- prefer not to answer

[Q43] {X} influences when I see, and the amount of time I spend with, my friends.

- 1 - I strongly disagree
- 2
- 3
- 4
- 5
- 6
- 7 - I strongly agree
- prefer not to answer

[Q44] {X} does not influence which of my friends I see.

- 1 - I strongly disagree
- 2
- 3
- 4
- 5
- 6
- 7 - I strongly agree
- prefer not to answer

[Q45] {X} does not influence the type of career I have.

- 1 - I strongly disagree
- 2
- 3
- 4
- 5
- 6
- 7 - I strongly agree
- prefer not to answer

[Q46] {X} influences or will influence how much time I devote to my career.

- 1 - I strongly disagree
- 2
- 3
- 4
- 5
- 6
- 7 - I strongly agree
- prefer not to answer

[Q47] {X} does not influence my chance of getting a good job in the future.

- 1 - I strongly disagree
- 2
- 3
- 4
- 5
- 6
- 7 - I strongly agree
- prefer not to answer

[Q48] {X} influences the way I feel about the future.

- 1 - I strongly disagree
- 2
- 3
- 4
- 5
- 6
- 7 - I strongly agree
- prefer not to answer

[Q49] {X} does not have the capacity to influence how I act in various situations.

- 1 - I strongly disagree
- 2
- 3
- 4
- 5
- 6
- 7 - I strongly agree
- prefer not to answer

[Q50] {X} influences and contributes to my overall happiness.

- 1 - I strongly disagree
- 2
- 3
- 4
- 5
- 6
- 7 - I strongly agree
- prefer not to answer

[Q51] {X} does not influence my present financial security.

- 1 - I strongly disagree
- 2
- 3
- 4
- 5
- 6
- 7 - I strongly agree
- prefer not to answer

[Q52] {X} influences how I spend my free time.

- 1 - I strongly disagree
- 2
- 3
- 4
- 5
- 6
- 7 - I strongly agree
- prefer not to answer

[Q53] {X} influences when I see {X} and the amount of time the two of us spend together.

- 1 - I strongly disagree
- 2
- 3
- 4
- 5
- 6
- 7 - I strongly agree
- prefer not to answer

[Q54] {X} does not influence how I dress.

- 1 - I strongly disagree
- 2
- 3
- 4
- 5
- 6
- 7 - I strongly agree
- prefer not to answer

[Q55] {X} influences how I decorate my home (e.g., apartment, house, dorm room, ...).

- 1 - I strongly disagree
- 2
- 3
- 4
- 5
- 6
- 7 - I strongly agree
- prefer not to answer

[Q56] {X} does not influence where I live.

- 1 - I strongly disagree
- 2
- 3
- 4
- 5
- 6
- 7 - I strongly agree
- prefer not to answer

[Q57] {X} influences what I watch on TV.

- 1 - I strongly disagree
- 2
- 3
- 4
- 5
- 6
- 7 - I strongly agree
- prefer not to answer

[Page break here]

Now we would like you to tell us how much {X} affects your future plans and goals. Using the 7-point scale below, please indicate the degree to which your future plans and goals are affected by {X} by clicking the appropriate scale. If an area does not apply to you (e.g., because you have no plans or goals in that area), click "1 - not at all".

[Q58] {X} affects my vacation plans.

- 1 - not at all
- 2
- 3
- 4
- 5
- 6
- 7 - a great extent
- prefer not to answer

[Q59] {X} affects my marriage plans.

- 1 - not at all
- 2
- 3
- 4
- 5
- 6
- 7 - a great extent
- prefer not to answer

[Q60] {X} affects my plans to have children.

- 1 - not at all
- 2
- 3
- 4
- 5
- 6
- 7 - a great extent
- prefer not to answer

[Q61] {X} affects my plans to make major investments (house, car, etc.).

- 1 - not at all
- 2
- 3
- 4
- 5
- 6
- 7 - a great extent
- prefer not to answer

[Q62] {X} affects my plans to join a club, social organization, church, etc.

- 1 - not at all
- 2
- 3
- 4
- 5
- 6
- 7 - a great extent
- prefer not to answer

[Q63] {X} affects my school-related plans.

- 1 - not at all
- 2
- 3
- 4
- 5
- 6
- 7 - a great extent
- prefer not to answer

[Q64] {X} affects my plans for achieving a particular financial standard of living.

- 1 - not at all
- 2
- 3
- 4
- 5
- 6
- 7 - a great extent
- prefer not to answer

Please, read each statement carefully, and click the answer that best corresponds to your agreement or disagreement with each statement.

[Q65] If {X} were feeling badly, my first duty would be to cheer him/her up.

- Not at all true; disagree completely


- Neither agree nor disagree


- Definitely true; agree completely
- Prefer not to answer

[Q66] I feel that I can confide in {X} about virtually everything.

- Not at all true; disagree completely


- Neither agree nor disagree


- Definitely true; agree completely
- Prefer not to answer

[Q67] I find it easy to ignore {X}'s faults.

- Not at all true; disagree completely


- Neither agree nor disagree


- Definitely true; agree completely
- Prefer not to answer

[Q68] I would do almost anything for {X}.

- Not at all true; disagree completely


- Neither agree nor disagree


- Definitely true; agree completely
- Prefer not to answer

[Q69] I feel very possessive toward {X}.

- Not at all true; disagree completely


- Neither agree nor disagree


- Definitely true; agree completely
- Prefer not to answer

[Q70] If I could never be with {X} I would feel miserable.

- Not at all true; disagree completely


- Neither agree nor disagree


- Definitely true; agree completely
- Prefer not to answer

[Q71] If I were lonely my first thought would be to seek {X} out.

- Not at all true; disagree completely


- Neither agree nor disagree


- Definitely true; agree completely
- Prefer not to answer

[Q72] One of my primary concerns is {X}'s welfare.

- Not at all true; disagree completely


- Neither agree nor disagree


- Definitely true; agree completely
- Prefer not to answer

[Q73] I would forgive {X} for practically anything.

- Not at all true; disagree completely


- Neither agree nor disagree


- Definitely true; agree completely
- Prefer not to answer

[Q74] I feel responsible for {X}'s well-being.

- Not at all true; disagree completely


- Neither agree nor disagree


- Definitely true; agree completely
- Prefer not to answer

[Q75] When I am with {X} I spend a good deal of time just looking at him (her).

- Not at all true; disagree completely


- Neither agree nor disagree


- Definitely true; agree completely
- Prefer not to answer

[Q76] I would greatly enjoy being confided in by {X}.

- Not at all true; disagree completely


- Neither agree nor disagree


- Definitely true; agree completely
- Prefer not to answer

[Q77] I would be hard for me to get along without {X}.

- Not at all true; disagree completely


- Neither agree nor disagree


- Definitely true; agree completely
- Prefer not to answer

[Page break here]

Please, read each statement carefully, and click the answer that best corresponds to your agreement or disagreement with each statement.

[Q78] When I am with {X} we are almost always in the same mood.

- Not at all true; disagree completely


- Neither agree nor disagree


- Definitely true; agree completely
- Prefer not to answer

[Q79] I think that {X} is unusually well adjusted.

- Not at all true; disagree completely


- Neither agree nor disagree


- Definitely true; agree completely
- Prefer not to answer

[Q80] I would highly recommend {X} for a responsible job.

- Not at all true; disagree completely


- Neither agree nor disagree


- Definitely true; agree completely
- Prefer not to answer

[Q81] In my opinion, {X} is an exceptionally mature person.

- Not at all true; disagree completely


- Neither agree nor disagree


- Definitely true; agree completely
- Prefer not to answer

[Q82] I have great confidence in {X}'s good judgment.

- Not at all true; disagree completely


- Neither agree nor disagree


- Definitely true; agree completely
- Prefer not to answer

[Q83] Most people would react very favorably to {X} after a brief acquaintance.

- Not at all true; disagree completely


- Neither agree nor disagree


- Definitely true; agree completely
- Prefer not to answer

[Q84] I think that {X} and I are quite similar to each other.

- Not at all true; disagree completely


- Neither agree nor disagree


- Definitely true; agree completely
- Prefer not to answer

[Q85] I would vote for {X} in a group election.

- Not at all true; disagree completely


- Neither agree nor disagree


- Definitely true; agree completely
- Prefer not to answer

[Q86] I think that {X} is one of those people who quickly wins respect.

- Not at all true; disagree completely


- Neither agree nor disagree


- Definitely true; agree completely
- Prefer not to answer

[Q87] I feel that {X} is an extremely intelligent person.

- Not at all true; disagree completely


- Neither agree nor disagree


- Definitely true; agree completely
- Prefer not to answer

[Q88] {X} is one of the most likable people I know.

- Not at all true; disagree completely


- Neither agree nor disagree


- Definitely true; agree completely
- Prefer not to answer

[Q89] {X} is the sort of person whom I myself would like to be.

- Not at all true; disagree completely


- Neither agree nor disagree


- Definitely true; agree completely
- Prefer not to answer

[Q90] It seems to me that it is very easy for {X} to gain admiration.

- Not at all true; disagree completely


- Neither agree nor disagree


- Definitely true; agree completely
- Prefer not to answer

Using the scale as a guide, select a number beside each statement to indicate how much you agree with it.

[Q91] My first impressions of people usually turn out to be right.

- 1 - not true
- 2
- 3
- 4 - somewhat true
- 5
- 6
- 7 - very true
- prefer not to answer

[Q92] It would be hard for me to break any of my bad habits.

- 1 - not true
- 2
- 3
- 4 - somewhat true
- 5
- 6
- 7 - very true
- prefer not to answer

[Q93] I don’t care to know what other people really think of me.

- 1 - not true
- 2
- 3
- 4 - somewhat true
- 5
- 6
- 7 - very true
- prefer not to answer

[Q94] I have not always been honest with myself.

- 1 - not true
- 2
- 3
- 4 - somewhat true
- 5
- 6
- 7 - very true
- prefer not to answer

[Q95] I always know why I like things.

- 1 - not true
- 2
- 3
- 4 - somewhat true
- 5
- 6
- 7 - very true
- prefer not to answer

[Q96] When my emotions are aroused, it biases my thinking.

- 1 - not true
- 2
- 3
- 4 - somewhat true
- 5
- 6
- 7 - very true
- prefer not to answer

[Q97] Once I’ve made up my mind, other people can seldom change my opinion.

- 1 - not true
- 2
- 3
- 4 - somewhat true
- 5
- 6
- 7 - very true
- prefer not to answer

[Q98] I am not a safe driver when I exceed the speed limit.

- 1 - not true
- 2
- 3
- 4 - somewhat true
- 5
- 6
- 7 - very true
- prefer not to answer

[Q99] I am fully in control of my own fate.

- 1 - not true
- 2
- 3
- 4 - somewhat true
- 5
- 6
- 7 - very true
- prefer not to answer

[Q100] It’s hard for me to shut off a disturbing thought.

- 1 - not true
- 2
- 3
- 4 - somewhat true
- 5
- 6
- 7 - very true
- prefer not to answer

[Q101] I never regret my decisions.

- 1 - not true
- 2
- 3
- 4 - somewhat true
- 5
- 6
- 7 - very true
- prefer not to answer

[Q102] I sometimes lose out on things because I can’t make up my mind soon enough.

- 1 - not true
- 2
- 3
- 4 - somewhat true
- 5
- 6
- 7 - very true
- prefer not to answer

[Q103] The reason I vote is because my vote can make a difference.

- 1 - not true
- 2
- 3
- 4 - somewhat true
- 5
- 6
- 7 - very true
- prefer not to answer

[Q104] My parents were not always fair when they punished me.

- 1 - not true
- 2
- 3
- 4 - somewhat true
- 5
- 6
- 7 - very true
- prefer not to answer

[Q105] I am a completely rational person.

- 1 - not true
- 2
- 3
- 4 - somewhat true
- 5
- 6
- 7 - very true
- prefer not to answer

[Q106] I rarely appreciate criticism.

- 1 - not true
- 2
- 3
- 4 - somewhat true
- 5
- 6
- 7 - very true
- prefer not to answer

[Q107] I am very confident of my judgments.

- 1 - not true
- 2
- 3
- 4 - somewhat true
- 5
- 6
- 7 - very true
- prefer not to answer

[Q108] I have sometimes doubted my ability as a lover.

- 1 - not true
- 2
- 3
- 4 - somewhat true
- 5
- 6
- 7 - very true
- prefer not to answer

[Q109] It’s all right with me if some people happen to dislike me.

- 1 - not true
- 2
- 3
- 4 - somewhat true
- 5
- 6
- 7 - very true
- prefer not to answer

[Q110] I don’t always know the reasons why I do the things I do.

- 1 - not true
- 2
- 3
- 4 - somewhat true
- 5
- 6
- 7 - very true
- prefer not to answer

[Q111] I sometimes tell lies if I have to.

- 1 - not true
- 2
- 3
- 4 - somewhat true
- 5
- 6
- 7 - very true
- prefer not to answer

[Q112] I never cover up my mistakes.

- 1 - not true
- 2
- 3
- 4 - somewhat true
- 5
- 6
- 7 - very true
- prefer not to answer

[Q113] There have been occasions when I have taken advantage of someone.

- 1 - not true
- 2
- 3
- 4 - somewhat true
- 5
- 6
- 7 - very true
- prefer not to answer

[Q114] I never swear.

- 1 - not true
- 2
- 3
- 4 - somewhat true
- 5
- 6
- 7 - very true
- prefer not to answer

[Q115] I sometimes try to get even rather than forgive and forget.

- 1 - not true
- 2
- 3
- 4 - somewhat true
- 5
- 6
- 7 - very true
- prefer not to answer

[Q116] I always obey laws, even if I’m unlikely to get caught.

- 1 - not true
- 2
- 3
- 4 - somewhat true
- 5
- 6
- 7 - very true
- prefer not to answer

[Q117] I have said something bad about a friend behind his or her back.

- 1 - not true
- 2
- 3
- 4 - somewhat true
- 5
- 6
- 7 - very true
- prefer not to answer

[Q118] When I hear people talking privately, I avoid listening.

- 1 - not true
- 2
- 3
- 4 - somewhat true
- 5
- 6
- 7 - very true
- prefer not to answer

[Q119] I have received too much change from a salesperson without telling him or her.

- 1 - not true
- 2
- 3
- 4 - somewhat true
- 5
- 6
- 7 - very true
- prefer not to answer

[Q120] I always declare everything at customs.

- 1 - not true
- 2
- 3
- 4 - somewhat true
- 5
- 6
- 7 - very true
- prefer not to answer

[Q121] When I was young I sometimes stole things.

- 1 - not true
- 2
- 3
- 4 - somewhat true
- 5
- 6
- 7 - very true
- prefer not to answer

[Q122] I have never dropped litter on the street.

- 1 - not true
- 2
- 3
- 4 - somewhat true
- 5
- 6
- 7 - very true
- prefer not to answer

[Q123] I sometimes drive faster than the speed limit.

- 1 - not true
- 2
- 3
- 4 - somewhat true
- 5
- 6
- 7 - very true
- prefer not to answer

[Q124] I never read sexy books or magazines.

- 1 - not true
- 2
- 3
- 4 - somewhat true
- 5
- 6
- 7 - very true
- prefer not to answer

[Q125] I have done things that I don’t tell other people about.

- 1 - not true
- 2
- 3
- 4 - somewhat true
- 5
- 6
- 7 - very true
- prefer not to answer

[Q126] I never take things that don’t belong to me.

- 1 - not true
- 2
- 3
- 4 - somewhat true
- 5
- 6
- 7 - very true
- prefer not to answer

[Q127] I have taken sick-leave from work or school even though I wasn’t really sick.

- 1 - not true
- 2
- 3
- 4 - somewhat true
- 5
- 6
- 7 - very true
- prefer not to answer

[Q128] I have never damaged a library book or store merchandise without reporting it.

- 1 - not true
- 2
- 3
- 4 - somewhat true
- 5
- 6
- 7 - very true
- prefer not to answer

[Q129] I have some pretty awful habits.

- 1 - not true
- 2
- 3
- 4 - somewhat true
- 5
- 6
- 7 - very true
- prefer not to answer

[Q130] I don’t gossip about other people’s business.

- 1 - not true
- 2
- 3
- 4 - somewhat true
- 5
- 6
- 7 - very true
- prefer not to answer

Here are a number of personality traits that may or may not apply to you. Please select a number next to each statement to indicate the extent to which you agree or disagree with that statement. You should rate the extent to which the pair of traits applies to you, even if one characteristic applies more strongly than the other.

[Q131] I see myself as: Extraverted, enthusiastic.

- 1 - disagree strongly
- 2 - disagree moderately
- 3 - disagree a little
- 4 - neither agree nor disagree
- 5 - agree a little
- 6 - agree moderately
- 7 - agree strongly
- prefer not to answer

[Q132] I see myself as: Critical, quarrelsome.

- 1 - disagree strongly
- 2 - disagree moderately
- 3 - disagree a little
- 4 - neither agree nor disagree
- 5 - agree a little
- 6 - agree moderately
- 7 - agree strongly
- prefer not to answer

[Q133] I see myself as: Dependable, self-disciplined.

- 1 - disagree strongly
- 2 - disagree moderately
- 3 - disagree a little
- 4 - neither agree nor disagree
- 5 - agree a little
- 6 - agree moderately
- 7 - agree strongly
- prefer not to answer

[Q134] I see myself as: Anxious, easily upset.

- 1 - disagree strongly
- 2 - disagree moderately
- 3 - disagree a little
- 4 - neither agree nor disagree
- 5 - agree a little
- 6 - agree moderately
- 7 - agree strongly
- prefer not to answer

[Q135] I see myself as: Open to new experiences, complex.

- 1 - disagree strongly
- 2 - disagree moderately
- 3 - disagree a little
- 4 - neither agree nor disagree
- 5 - agree a little
- 6 - agree moderately
- 7 - agree strongly
- prefer not to answer

[Q136] I see myself as: Reserved, quiet.

- 1 - disagree strongly
- 2 - disagree moderately
- 3 - disagree a little
- 4 - neither agree nor disagree
- 5 - agree a little
- 6 - agree moderately
- 7 - agree strongly
- prefer not to answer

[Q137] I see myself as: Sympathetic, warm.

- 1 - disagree strongly
- 2 - disagree moderately
- 3 - disagree a little
- 4 - neither agree nor disagree
- 5 - agree a little
- 6 - agree moderately
- 7 - agree strongly
- prefer not to answer

[Q138] I see myself as: Disorganized, careless.

- 1 - disagree strongly
- 2 - disagree moderately
- 3 - disagree a little
- 4 - neither agree nor disagree
- 5 - agree a little
- 6 - agree moderately
- 7 - agree strongly
- prefer not to answer

[Q139] I see myself as: Calm, emotionally stable.

- 1 - disagree strongly
- 2 - disagree moderately
- 3 - disagree a little
- 4 - neither agree nor disagree
- 5 - agree a little
- 6 - agree moderately
- 7 - agree strongly
- prefer not to answer

[Q140] I see myself as: Conventional, uncreative.

- 1 - disagree strongly
- 2 - disagree moderately
- 3 - disagree a little
- 4 - neither agree nor disagree
- 5 - agree a little
- 6 - agree moderately
- 7 - agree strongly
- prefer not to answer

Thank you! You're almost done, just answer these two questions and the HIT is done.

[Q141] To what extent have you participated in other studies involving similar questionnaires on MTurk before taking this HIT? Take a guess if you are not sure.

- never
- 1-10
- 11-20
- 21-50
- more than 50

[Q142] What is your nationality?

- USA
- Other ____________________
