# Supplementary material for: Measuring the Closeness of Relationships: A Comprehensive Evaluation of the 'Inclusion of the Other in the Self' Scale
Source: PLoS One. 2015 Jun 12;10(6):e0129478. doi: 10.1371/journal.pone.0129478 (PMC4466912; doi:10.1371/journal.pone.0129478)
Supplement: S2 Text — (DOCX) [file pone.0129478.s010.docx]

**S2 Text**

Details of the Principal Component Analysis

pca we rci_tot love_tot like_tot pam_tot sci_tot

Principal components/correlation Number of obs = 431

Number of comp. = 6

Trace = 6

Rotation: (unrotated = principal) Rho = 1.0000

--------------------------------------------------------------------------

Component | Eigenvalue Difference Proportion Cumulative

-------------+------------------------------------------------------------

Comp1 | 4.50311 3.94969 0.7505 0.7505

Comp2 | .553419 .179957 0.0922 0.8428

Comp3 | .373462 .101276 0.0622 0.9050

Comp4 | .272185 .083777 0.0454 0.9504

Comp5 | .188408 .0789881 0.0314 0.9818

Comp6 | .10942 . 0.0182 1.0000

--------------------------------------------------------------------------

Principal components (eigenvectors)

----------------------------------------------------------------------------------------

Variable | Comp1 Comp2 Comp3 Comp4 Comp5 Comp6 | Unexplained

-------------+------------------------------------------------------------+-------------

we | 0.4169 -0.1978 -0.1918 -0.7042 0.4945 0.0998 | 0

rci_tot | 0.3831 -0.4585 0.7363 0.2324 0.0687 0.2054 | 0

love_tot | 0.4462 0.0256 0.0844 -0.1372 -0.3673 -0.7996 | 0

like_tot | 0.3501 0.8608 0.2818 0.0271 0.1621 0.1735 | 0

pam_tot | 0.4119 -0.0824 -0.4770 0.6554 0.3914 -0.1154 | 0

sci_tot | 0.4338 -0.0476 -0.3273 -0.0317 -0.6606 0.5148 | 0

----------------------------------------------------------------------------------------

. screeplot, yline(1) graphregion(fcolor(white))
